# Salicylate-Induced Suppression of Electrically Driven Activity in Brain Slices from the Auditory Cortex of Aging Mice

**DOI:** 10.3389/fnagi.2017.00395

**Published:** 2017-12-12

**Authors:** Minoru Namikawa, Ayaka Sano, Takashi Tateno

**Affiliations:** Department of Bioengineering and Bioinformatics, Graduate School of Information Science and Technology, Hokkaido University, Sapporo, Japan

**Keywords:** auditory brainstem response, auditory cortex, GABA, local field potential, salicylate, slice preparations

## Abstract

The prevalence of tinnitus is known to increase with age. The age-dependent mechanisms of tinnitus may have important implications for the development of new therapeutic treatments. High doses of salicylate can be used experimentally to induce transient tinnitus and hearing loss. Although accumulating evidence indicates that salicylate induces tinnitus by directly targeting neurons in the peripheral and central auditory systems, the precise effect of salicylate on neural networks in the auditory cortex (AC) is unknown. Here, we examined salicylate-induced changes in stimulus-driven laminar responses of AC slices with salicylate superfusion in young and aged senescence-accelerated-prone (SAMP) and -resistant (SAMR) mice. Of the two strains, SAMP1 is known to be a more suitable model of presbycusis. We recorded stimulus-driven laminar local field potential (LFP) responses at multi sites in AC slice preparations. We found that for all AC slices in the two strains, salicylate always reduced stimulus-driven LFP responses in all layers. However, for the amplitudes of the LFP responses, the two senescence-accelerated mice (SAM) strains showed different laminar properties between the pre- and post-salicylate conditions, reflecting strain-related differences in local circuits. As for the relationships between auditory brainstem response (ABR) thresholds and the LFP amplitude ratios in the pre- vs. post-salicylate condition, we found negative correlations in layers 2/3 and 4 for both older strains, and in layer 5 (L5) in older SAMR1. In contrast, the GABAergic agonist muscimol (MSC) led to positive correlations between ABR thresholds and LFP amplitude ratios in the pre- vs. post-MSC condition in younger SAM mice from both strains. Further, in younger mice, salicylate decreased the firing rate in AC L4 pyramidal neurons. Thus, salicylate can directly reduce neural excitability of L4 pyramidal neurons and thereby influence AC neural circuit activity. That we observed age-dependent effects of salicylate and varied GABAergic sensitivity in the AC among mouse strains with hearing loss implies that potential therapeutic mechanisms for tinnitus may operate differently in young vs. aged subjects. Therefore, scientists developing new therapeutic modalities for tinnitus treatment should consider using both aged and young animals.

## Introduction

Aging is associated with an increased prevalence of tinnitus. Specifically, the incidence of tinnitus in humans increases monotonically up to approximately 70 years of age (Møller, 2011). Therefore, examining the influence of aging may lead to new therapeutic mechanisms for tinnitus, for instance, treatments that are specialized for patients from different age groups. High doses of sodium salicylate (SS), which is the active ingredient in aspirin, have been found to induce hearing loss and tinnitus in humans (Myers et al., 1965; McFadden et al., 1984; Brien, 1993; for a review see Cazals, 2000) and animals (Yang et al., 2007; Chen et al., 2013; Radziwon et al., 2015). Further, salicylate is widely used to study the behavioral, anatomical, physiological and perceptual characteristics of the auditory system in animal models (for a review see Sheppard et al., 2014).

Although SS-induced tinnitus is widely thought to have a peripheral origin (Guitton et al., 2003; Ruel et al., 2008), accumulating experimental data show that SS induces tinnitus by directly targeting neurons in the central auditory system in animal models (Brennan and Jastreboff, 1991; Lobarinas et al., 2004; Yang et al., 2007; Kizawa et al., 2010; for a review see Eggermont, 2016). In addition, although many central auditory structures have been implicated in tinnitus, recent studies have reported that the auditory cortex (AC) plays an important role. However, an important challenge in studying the central mechanisms of tinnitus has been the distinction of peripheral from central changes in the whole auditory pathway. Therefore, brain slice preparations may be useful in exploring the functional roles of specific local networks because they allow the isolation of subcircuits within the auditory system.

In a previous *in vitro* study, Wang et al. (2006) reported that 1.4 mM SS depressed current-evoked and spontaneous miniature inhibitory postsynaptic currents recorded from pyramidal neurons in layer 2/3 (L2/3) of AC slices obtained from very young (postnatal 12- to 15-day-old) rats (Wang et al., 2006). This indicates that the SS-induced reduction in inhibitory postsynaptic currents contributed to the increased excitability of the AC *in vitro*. In addition, Basta et al. (2008) reported that, in extracellular single unit recordings, 1.4 mM SS superfusion modulated spontaneous neuronal firing rates in the primary AC (A1) in brain slices from young (20- to 45-day-old) rats; 28.6%, 42.9% and 28.5% of examined neurons exhibited increased, decreased, and unchanged firing rates, respectively (Basta et al., 2008). Su et al. (2009) reported that in whole-cell patch-clamp recordings in young (postnatal 12- to 19-day-old) rat AC slices, 1.4 mM SS depressed current-evoked firing in layer II/III fast-spiking interneurons, which are putatively GABAergic and inhibitory, but not in excitatory pyramidal neurons in the same layer. This suggests that SS increases excitability through disinhibition in the central auditory system, leading to tinnitus (Su et al., 2009). However, the influence of SS on whole local neuronal networks in the AC *in vitro* is unknown.

Senescence-accelerated mice (SAM) are an important biogerontological resource in aging research worldwide (Takeda et al., 1981, 2013; Hosokawa et al., 1984; Takeda, 1999). SAM include senescence-accelerated-prone (SAMP) and -resistant (SAMR) mice. Compared with SAMR1, SAMP1 represent a more suitable model of presbycusis because they exhibit progressive hearing loss with an earlier onset (Hosokawa et al., 2013). The direct association between aging and tinnitus can be examined using SAM combined with a tinnitus inducing agent such as SS. However, no previous studies have combined these two variables. Recently, to examine age-related differences in SAM, we extracellularly recorded spatiotemporal laminar profiles in AC slices using a planar multielectrode array (MEA) substrate, which enabled us to present electric local stimulation in specific cortical layers (Yamamura et al., 2017). In terms of the association between cortical activity and aging characteristics among SAM strains, we found lower variability in current source density (CSD) patterns of stimulus-driven responses in older SAMP1 mice. This may reflect earlier age-related changes in local AC networks in SAMP1 compared with SAMR1 mice.

Here, to extend previous investigations of GABAergic dysfunction in animal models of tinnitus (Wang et al., 2011; Llano et al., 2012), we examined SS-induced changes in stimulus-driven laminar responses in SAM AC slices, and pharmacologically manipulated the local AC circuit *in vitro*. In particular, we sought to answer the following questions using the two SAM strains: (i) In response to current stimulation in AC slices, does SS induce characteristic spatiotemporal local field potential (LFP) profiles in a cortical layer-dependent manner?; (ii) Do young and aged mice from different SAM strains vary in terms of SS-induced LFP responses?; (iii) Are SS-induced responses modulated according to the sensitivity of the peripheral auditory system?; (iv) How are spatiotemporal LFP response profiles influenced by the application of SS in the presence/absence of a GABAergic agonist/antagonist? Finally, we discuss AC local network properties in the context of tinnitus and aging in the different SAM strains. We speculate that examining the cortical mechanisms induced by SS in young vs. aged mice could lead to new human treatments aimed at altering cortical activity to relieve tinnitus and reduce dysfunction in the AC.

## Materials and Methods

All animal experiments described below were carried out in accordance with the National Institute of Health Guidelines for the Care and Use of Laboratory Animals and with the approval of the Institutional Animal Care and Use Committee of Hokkaido University. We used SAM-R1 and -P1 (Takeda et al., 1981; Takeda, 2009) as well as C57BL/6J mice. Three-week old mice of all three strains were obtained from Japan SLC Inc. (Shizuoka, Japan), and maintained until they reached the appropriate age for experiments. Previous studies using auditory brainstem responses (ABRs) to assess mouse hearing impairments reported that SAMP1 mice aged more than 12 months (52 weeks) exhibited hearing loss due to age-related changes in spiral ganglion neuron density (Saitoh et al., 1994; Takeda, 2009). In C57BL/6J mice, progressive hearing loss in the peripheral and central nervous systems reportedly begins at about 2 months of age. This hearing loss affects the highest spectral frequencies first, and then increases in severity between 12–15 months of age and spreads to include lower frequencies (Hunter and Willott, 1987; Keithley et al., 2004; Ison et al., 2007).

### ABR Recording

Before the *in vitro* electrophysiological experiments, we evaluated the auditory properties of the mice via ABR recording. For ABR testing, mice were first anesthetized with a mixture of midazolam (10 mg/kg i.p.; Astells Pharma, Japan) and xylazine (12 mg/kg i.p.; Bayer AG, Germany) in saline (Inaoka et al., 2011; Tateno et al., 2013; Noto et al., 2016). Anesthesia adequacy was confirmed by the absence of reflexes to toe pinches. We performed ABR testing in a sound-attenuating, electrically shielded chamber (Noto et al., 2016). For recording, three subdermal needle electrodes were inserted under the skin. The active electrode was positioned on the vertex of each mouse. The reference and ground electrodes were placed in the patch of bare skin behind the pinna overlying the bulla (Ingham et al., 2011; Tateno et al., 2013). ABR recordings were obtained from both the left and right ear in turn. Sound stimulation was delivered through an earphone to the ear of the animal. Before each set of experiments, using a sound level meter (Type 2636, Brüel and Kjaer, Denmark) with a 1/4-inch microphone (Type 4939-L-002, Brüel and Kjaer, Denmark), the sound-delivery system was calibrated. Sound signals were generated digitally at a rate of 100 kHz, processed via digital-to-analog conversion, attenuated, and amplified using TDT system 3 hardware (RP2.1, PA5 and SA1) and software RPvdsEX (Tucker-Davis Technologies, Alachua, FL, USA). Sound stimuli were positive or negative monophasic clicks with a 5-ms duration, ranging in sound level from 5 dB to 80 dB sound pressure level (SPL) in 5-dB steps. During ABR recording at a sampling rate of 44.8 kHz, 500 trials with positive or negative click sounds were alternately presented at a rate of 21 Hz. One set of trials took about 6.3 min. After recording, the responses were averaged, and the threshold was determined. The threshold was defined as the lowest click intensity that evoked a clear and characteristic deflection (typical wave I to V) in the average ABR signal that was greater than three times the standard deviation (SD) of the background noise in the recorded signals. Wave I voltage is known to originate from the cochlea and/or compound action potential of auditory nerve, and the next four waves (waves II to V) arise from ascending generators in the auditory midbrain: cochlear nucleus, contralateral superior olivary complex, lateral lemniscus, and contralateral lateral inferior colliculus (IC), respectively (Henry, 1979; for review, Parham et al., 2001).

### Brain Slice Preparation

All electrophysiological *in vitro* recordings were carried out within 3 days of the ABR recordings. Our method of brain slice preparation has been described previously (Yamamura and Tateno, 2017; Yamamura et al., 2017). Briefly, a mouse was deeply anesthetized with halothane and then decapitated, and a brain block containing the AC was rapidly removed. The brain block was submerged in chilled artificial cerebrospinal fluid (ACSF) saturated with 95% O_2_ and 5% CO_2_ mixed gas. The ACSF contained (in mM): 119 NaCl, 26.2 NaHCO_3_, 2.5 KCl, 2.5 CaCl_2_, 1.3 MgSO_4_, 1.0 NaH_2_PO_4_ and 11.0 D-glucose (pH = 7.4). Then, slices were cut with a tissue slicer (Linear Slicer Pro7, D.S.K., Japan) in the chilled ACSF bubbled with mixed gas. To record LFPs and membrane action potentials from the mouse brain *in vitro*, 400-μm-thick coronal A1 slices were prepared (see below). In the slices, the distance of each coronal section from Bregma along the rostral/caudal axis was defined in the following way. A digitized atlas (Franklin and Paxinos, 2008) containing drawings of coronal sections through the mouse brain was consulted. We prepared the coronal section that best matched the drawing at 2.70–2.80 mm caudal to Bregma (Figures 53 and 54 in the atlas). Thus, we initially obtained two or three slices from one animal and then separated each slice into left and right pieces. Therefore, we acquired 4–6 pieces of AC slices from each animal. The electrophysiological recordings were carried out in a submerged-type holding chamber at 28°C in a water bath, and the slices were recovered for at least 2.5 h before the recordings.

### Multisite Extracellular Recording in AC Slices

For multisite extracellular recordings, we used SAM mice in two age groups: 3–5 months (group A or younger group) and 12–14 months (group B or older group; Table 1). In several experiments, we compared the results obtained from the younger SAM (group A) to those obtained from age-matched C57BL/6J mice (3–5 months; Table 1).

**Table 1:** Summary of tested mice in the auditory brainstem response (ABR) recordings and slices in the extracellular multielectrode array (MEA) recordings.

Mouse strain	Group	Age group	Age in month*	Number of animals	Number of slices
SAMR1	A	Younger	4.1 ± 0.2	9	17
	B	Older	12.8 ± 0.1	12	18
SAMP1	A	Younger	4.1 ± 0.2	9	13
	B	Older	13.0 ± 0.2	11	16
C57BL/6J	A	Younger	4.0 ± 0.9	13	15

*Data are shown as the mean ± SEM.

Our methods for multisite extracellular recording mirrored those in previous reports (Yamamura et al., 2017). Briefly, all brain slice recordings were performed in ACSF with mixed gas. The ACSF solution was administered from an incubator at the top of the recording chamber (APC-30, Asteck Co., Japan) maintained at 28.0°C. This temperature, which was lower than the average mouse body temperature, has been found to be suitable for long recording durations. We also chose to use this temperature so that our results would be comparable to those from previous studies (Wang et al., 2006; Su et al., 2009; Yamamura et al., 2017). For recording, slices were plated on an MEA substrate (MED-P515A, Alpha MED Scientific, Japan) and covered with a piece of nylon mesh and a stainless slice anchor. Each of the 64 recording sites in the array covered a 50 × 50 μm square area. The distance between the centers of adjacent sites was 150 μm. Aerated ACSF at 28°C was continuously circulated through the submerged recording chamber at a rate of 1.2 mL/min. In a typical recording, the recording electrode sites were located at cortical layers in the A1 (Figure 2A). In localizing the AC, we used the hippocampus and rhinal fissure as landmarks (Franklin and Paxinos, 2008). For electrical stimulation, we selected a single stimulation electrode site in the AC layer 4 (L4), unless otherwise mentioned. The columns in the recording sites were mostly vertical to the surface of the pia matter. Each column containing a stimulation site is hereafter referred to as a vertical “on-line” column (Figure 2B). Throughout the experiments described here, we used a fixed small stimulus current intensity ranging from 15 μA to 20 μA. The intensity was usually set at 20%–30% of the stable level in terms of the input-output relationship between the input current intensities and evoked LFPs. We confirmed that the stimulation intensity did not evoke global activity propagation over the whole slice, and that local activity was restricted to the region around the stimulation site (Figure 2B). In the control condition, we selected one stimulation site in L4 and confirmed the stability of the responses before applying a test stimulus (a single 200-μs-width bipolar pulse; 100 μs negative current, followed by 100 μs positive current) every 20–30 s for 70–140 min. Our experimental long-term potentiation/depression recording system was developed to enable experimenters to obtain stable evoked responses over 4 h (Yamamura and Tateno, 2017). During preparations for the experiment, we confirmed that evoked LFP responses to small current-pulse stimulation in the control condition were very stable over 2 h. We recorded evoked LFP responses in each slice simultaneously at a sampling rate of 20 kHz. The signals were filtered from 1 Hz to 10 kHz.

**Figure 1:**
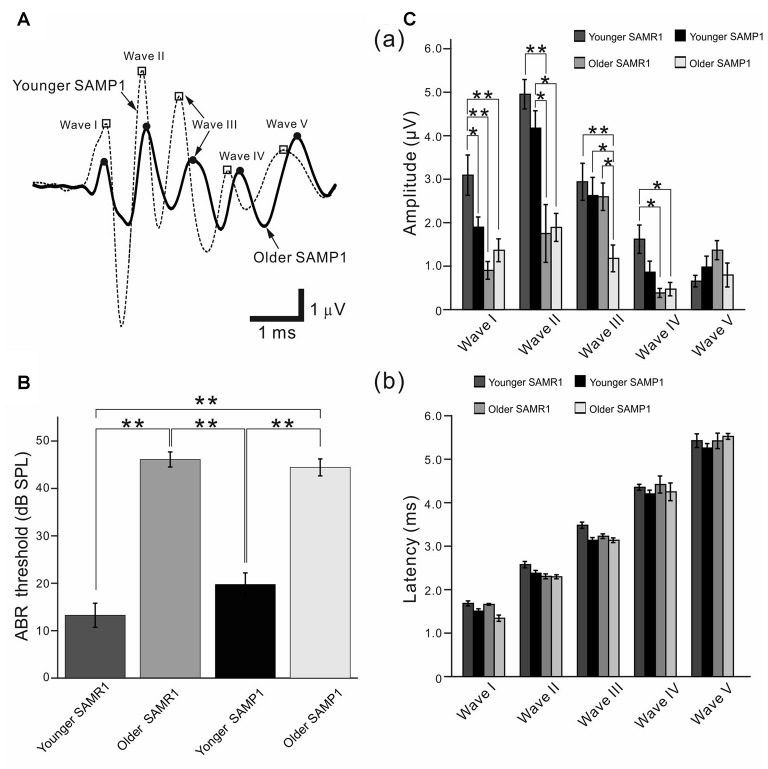
Auditory brainstem response (ABR) recordings from senescence-accelerated mice (SAM) strains. **(A)** Typical examples of average ABR waveforms obtained from younger and older SAMP1 mice in response to click sound stimuli. Only the waveforms from representative SAMP1 mice are shown because those obtained from SAMR1 mice were quite similar. The waveforms from the younger and older mouse groups were respectively averaged for over 500 click sound stimuli at 65 dB sound pressure level (SPL; see “Materials and Methods” section). Open squares (□) and filled circles (●) mark the local peaks of ABR waves I to V from left to right. **(B)** ABR thresholds for the click sound stimuli were compared among the younger and older SAM-R1 and -P1 mice. Asterisks (**) represent *P* < 0.01 by the Tukey-Kramer multiple comparison test. **(C)** Local peak amplitudes in **(a)** and peak latency in **(b)**, which were quantified from the ABR recordings in response to the click stimuli at 65 dB SPL among the four mouse groups. *P < 0.05 and **P < 0.01 by the Tukey-Kramer multiple comparison test.

**Figure 2:**
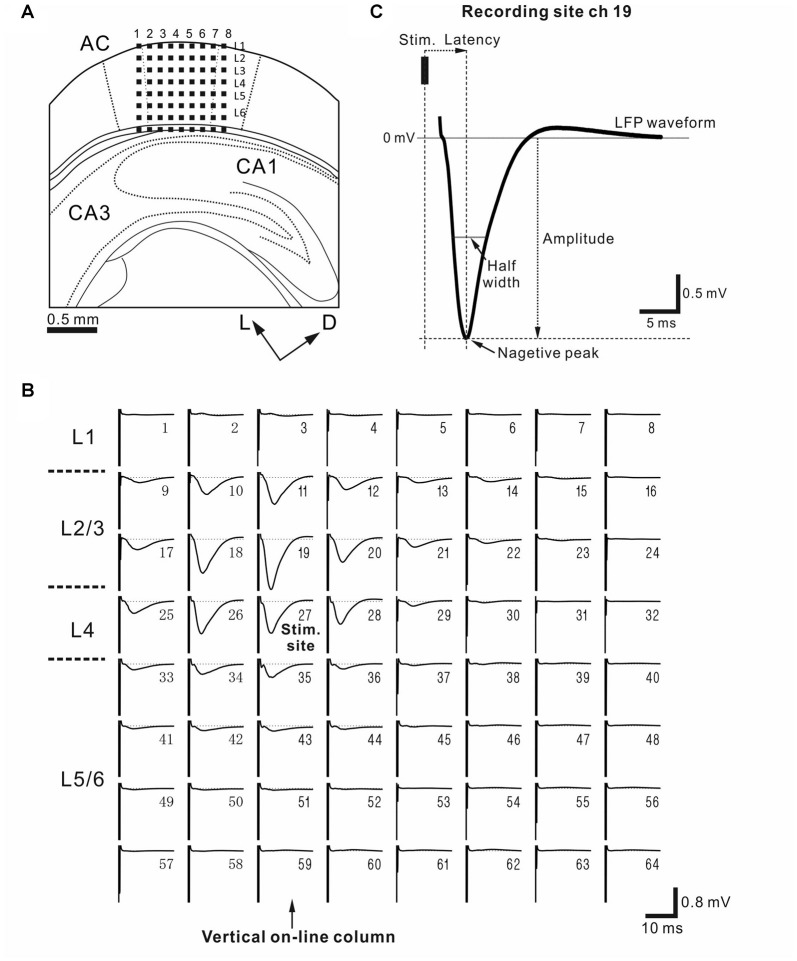
**(A)** Schematic diagram of a cortical coronal slice on a multielectrode array (MEA) substrate. Evoked local field potentials (LFPs) were recorded extracellularly from a slice of the mouse auditory cortex (AC) on MEA substrate with 64-channel (ch) electrodes. Small dark squares represent the 64 electrodes. The size of each electrode was 50 × 50 μm^2^, and the inter-electrode interval was 150 μm. The AC was schematically located according to the standard mouse brain atlas (Franklin and Paxinos, 2008). Dorsal and lateral directions are represented by two arrows labeled D and L, respectively. Each column is identified by a number (1–8) from left to right. **(B)** Representative LFP waveforms in a coronal AC slice. Each waveform was obtained by averaging 10 trials. A stimulating electrode (ch 27) at the 3rd row from the top and the 3th column from the left was situated in L4 or the border between L4 and L3, and the stimulation site was indicated by “stim. site”. During recording, the stimulation current intensity was 15 μA (see “Materials and Methods” section). The numbers in the upper and right regions of the subplots represent the channel numbers of the 64 electrodes. The onset timing of the stimulus is identified by a large artifact on the left side in each LFP plot. Columns with recording sites vertical to the stimulus site are referred to as vertical “on-line” columns, and are indicated by an arrow. **(C)** Typical evoked average waveform illustrating the characteristic properties of LFP: peak amplitude, peak latency and half width of the peak of the LFP. The LFP had a small positive peak following a large negative peak. The recording and stimulation sites were chs 19 and 27, respectively.

To electrically characterize evoked LFP responses at a vertical on-line site in each slice, we analyzed the largest average negative-going LFP elicited by test stimuli (10 trials) in L4 (Figure 2C). In the LFP recording, response peak amplitude (*A*) was defined as the absolute voltage of the largest negative peak from the baseline (i.e., 0 mV) evoked by the stimulation. The amplitude ratio before and after perfusion of SS was defined as *A*_SS_/*A*_ctl_, where *A*_SS_ and *A*_ctl_ represent the response amplitudes during SS application and in the control condition, respectively. The response latency was defined as the time interval from the stimulation onset to the largest negative peak (Figure 2B). Finally, the half width was defined as the time interval between the two time points corresponding to half peak amplitude (Figure 2C). If present, other drug effects on LFP properties were characterized according to amplitude, latency, and half width. Further, to characterize the time course of peak amplitude changes over time, the decay time constant (τ_d_) during drug application was defined as the time interval from the application onset to the time point when the amplitude was firstly reduced below 63.2% (100 × (1.0 − 1.0/e)%) of the initial stable level.

We digitally imaged the locations of all recording positions on the multielectrode substrate before and after recording, noted at the time of the experiment, and correlated histologically with the laminar positions, which were histologically determined via Nissl staining with cresyl violet (Yamamura et al., 2017). Additionally, after each recording, we evaluated the stimulation positions on the electrodes and cortical layers according to characteristic CSD profiles, specifically, the positions of current sinks and sources (see Supplementary Material Appendix A; Yamamura et al., 2017). The thickness of the cortical layers was consistent with previous research (DeFelipe et al., 2002).

### Whole-Cell Patch-Clamp Recording and Cell-Type Identification

We only used young adults of SAM and C57BL/6J mice (6–11 weeks in all strains) for whole-cell patch-clamp recordings because it was not possible to obtain stable recordings from older mice. The neurons in L4 of the AC slices were visually identified based on their morphology via an upright microscope (Olympus BW60WI, Olympus Japan, Japan) equipped with an infrared CCD camera (C3077-78, Hamamatsu Photonics, Japan). Whole-cell patch-clamp recordings were carried out at 26–28°C with patch pipettes (4–6 MΩ) pulled (Sutter P-97, Sutter, Atlanta, GA, USA) from borosilicate capillaries (Clark GC150F, Warner Instruments, Hamden, CT, USA). The pipettes were filled with a standard intracellular solution (in mM): 105 K-gluconate, 30 KCl, 10 HEPES, 10 phosphocreatine Na_2_, 4 ATP-Mg, 0.3 Na-GTP, and 5 mg/ml biocytin, balanced to pH 7.3 with KOH (Stuart and Sakmann, 1995; Tateno and Robinson, 2007). When the whole-cell patch-clamp recording configuration was established, the recording mode was switched from voltage-clamp to current-clamp. The slices were perfused continuously with ACSF solution at a flow rate of 1.2 ml/min during recordings. Somatic patch-pipette recordings were performed with a Multiclamp 700B amplifier (Molecular Devices, Sunnyvale, CA, USA) in current-clamp and voltage-clamp modes, correcting for the pre-nulled liquid junction potential. Only neurons with a resting potential (RP) that ranged from −80 mV to −50 mV and was stable for over 20 min were considered healthy. Signals were filtered at 10 kHz and sampled with a 16-bit resolution at 20 kHz through an AD converter (PCI-6052E, National Instruments, Austin, TX, USA).

To evoke a stable train of firing, we injected a 1-s suprathreshold depolarizing current into the recorded cells every 10 or 20 s. Following a stable baseline recording, the brain slice was superfused with ACSF containing a pharmacological agent and substantially washed out. To characterize action potential properties, we defined a number of parameters including spike amplitude, afterhyperpolarization (AHP), spike half width, and adaptation ratio (AR). For action potentials evoked by the 1-s current steps from a resting membrane potential, we used the third action potential from stimulation onset to characterize the shape parameters of regular spiking cells. As we observed intrinsic-burst-like firing in two cells, we used the second regular action potential observed after termination of burst firing. We measured spike amplitude as the difference between the peak and the threshold of the action potential (Tateno et al., 2004). Spike threshold was defined as the potential at which the first derivative of the voltage waveform exceeded five times its baseline SD. The AHP was measured as the difference between the spike threshold and voltage minimum following the action potential peak. The spike half width was measured at half the spike amplitude. Spike times were measured as the points at which the voltage increased above zero with respect to the membrane potential. Current strength usually progressively increased or decreased in small (10, 20, or 50 pA) steps. To construct frequency-current relationships, we plotted the initial frequency and steady-state firing rate as a function of the injected current intensity. We computed the maximum firing rate of a neuron using the number of spikes per trial at the highest current strength before the depolarization block. The AR of the action potentials was defined as the ratio of the first inter-spike interval after the onset of a 1-s current injection to that immediately before the offset of the injection. To examine membrane input resistance under the whole-cell current-clamp mode, negative current steps (duration, 500 ms) ranging from −80 pA to −30 pA with a 10-pA step were applied to neurons, and the input resistance was calculated from changes of the membrane voltage.

Our cell staining method was based on the study reported previously (Tateno and Robinson, 2011). Briefly, after recording, slices were rinsed three times in 0.1 M phosphate buffered saline (PBS; pH 7.4) and placed in cryoprotectant (30% sucrose in 0.05 M phosphate buffer at pH 7.4) for 2 h. Slices were then subjected to 1–3 cycles of rapid freezing in liquid nitrogen followed by thawing in cryoprotectant for 2 h to maximize penetration of streptavidin. All slices were then rinsed in PBS and soaked in 5% bacto-agar solution. If necessary, slices were resectioned at 50 μm using a vibratome (Leica VT1000, Leica Microsystems, Nussloch, Germany), to improve staining. Sections were then incubated in 0.2% Alexa Fluor 594-conjugated streptavidin (S-11227, Thermo Fisher Scientific, Waltham, MA, USA) for 1 h. Incubations were performed on free-floating sections at room temperature under gentle agitation in TBS. Finally, sections were rinsed and mounted on glass slides. Fluorescent images of recorded and biocytin-filled neurons were obtained using a confocal imaging system (Olympus FV-1000, Olympus, Japan). We traced the neuronal morphology of the stained cells via Neurolucida software (MicroBrightField, Williston, VT, USA).

To examine two major cell types in L4 neurons, we used a similar method reported in previous studies (Su et al., 2009). First, a video monitor connected to an upright microscope equipped with an infrared CCD camera, and the laminar structures and cell shapes in the AC slices were visualized. In L4, neurons with a triangular soma and apical dendrite approaching the pia were identified as putative pyramidal neurons (Figure 12). Neurons with a round or oval soma and multipolar dendrites were identified as putative fast-spiking interneurons (Supplementary Figure S3). We identified two typical neuronal types on the basis of their characteristic properties with respect to current-injected firing and the waveforms of individual action potentials. We applied the following criteria: (1) After the onset of a 1-s depolarizing current, we classified cells with apparent firing adaptation as pyramidal neurons and those with less firing adaptation as fast-spiking interneurons. (2) We classified cells that fired with a lower saturating rate (≤35 Hz) as pyramidal neurons and those that fired with a higher saturating rate as fast-spiking interneurons. (3) Pyramidal neurons had a lower AHP amplitude compared with fast-spiking interneurons. (4) Pyramidal neurons had broader action potential widths compared with fast-spiking interneurons. (5) After recording, we stained the cells and confirmed the morphology. Although we identified two neuronal types, we only analyzed data from pyramidal neurons in this study. Because the mouse AC contains a number of neuronal subtypes, we examined data from cells that could be clearly identified as pyramidal neurons (Xu et al., 2010; Ji et al., 2016).

**Figure 3:**
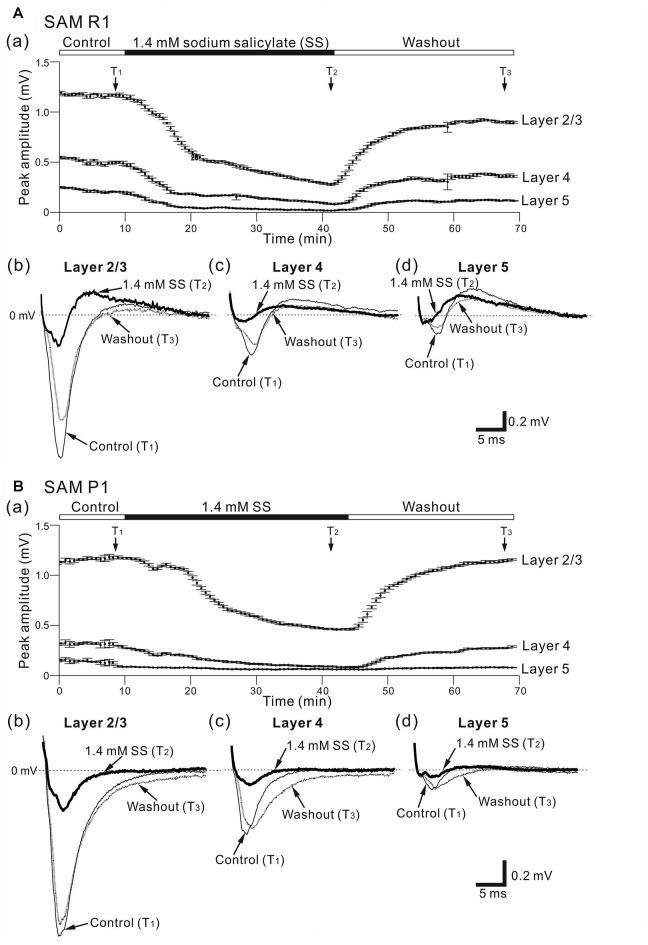
Effects of sodium salicylate (SS) on cortical LFPs evoked by stimulation at one site in L4. **(A)** Representative example of an older SAMR1 mouse (13-month old). In **(a)**, superfusion of 1.4-mM SS decreased negative peak LFP amplitudes (T_2_) in the three layers, compared with control (T_1_) or after the drug was washed out (T_3_). The durations of the three (pre-SS, post-SS and washout) conditions are given in the back and white bars on the top of the panel. Typical LFP responses observed in L2/3 **(b)**, L4 **(c)** and L5 **(d)** at the three time points (T_1_, T_2_ and T_3_) are illustrated. **(B)** Similarly, a representative example of an older SAMP1 mouse (13-month old). In **(a)**, superfusion of 1.4 mM SS also decreased negative peak LFP amplitudes (T_2_) compared with control (T_1_) or after the drug was washed out (T_3_). Typical LFP responses observed in L2/3 **(b)**, L4 **(c)** and L5 **(d)** at the three time points (T_1_, T_2_ and T_3_) are illustrated.

**Figure 4:**
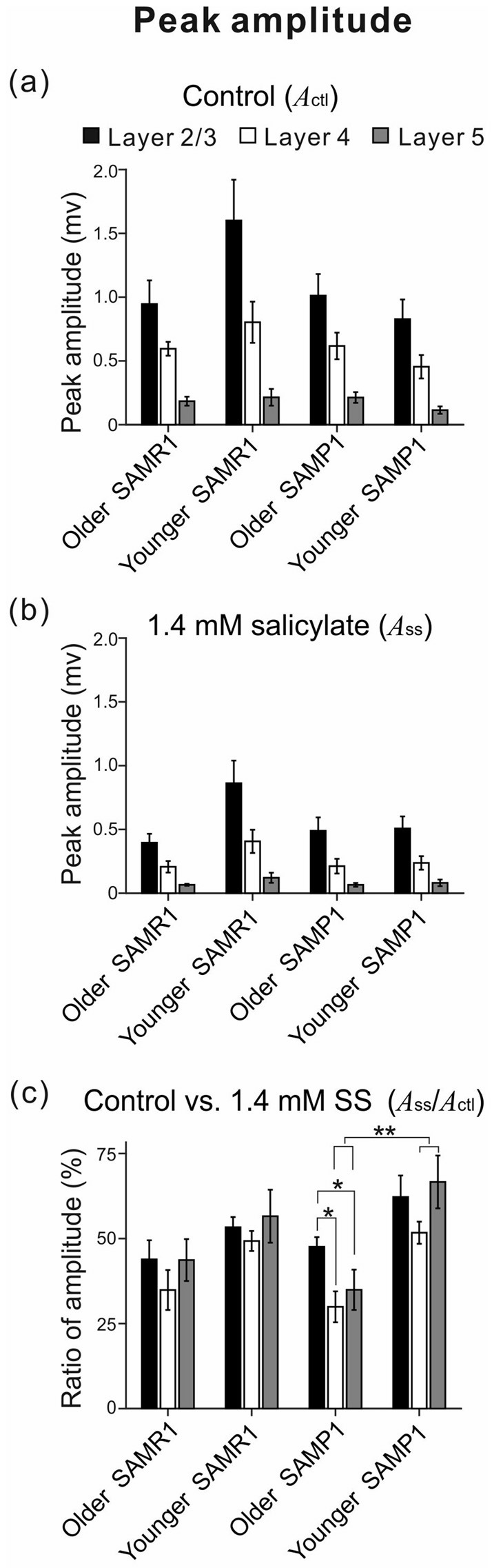
Among the four mouse groups (younger and older SAM-R1 and -P1), the peak amplitudes of stimulus-driven LFPs in the three layers (L2/3, L4 and L5) are shown before and after the SS superfusion. Peak amplitudes (*A*_ctl_ and *A*_SS_) in L2/3 (black), L4 (white) and L5 (gray) among the mouse groups are plotted under the control condition in **(a)** and the 1.4 mM SS superfusion condition in **(b)**. The ratios of the amplitudes (*A*_SS_/*A*_ctl_) among the four mouse groups are also illustrated in **(c)**. Error bars represent standard error of the mean (SEM). Data are shown as mean ± SEM. *P < 0.05 and **P < 0.01 by the Tukey-Kramer multiple comparison test.

**Figure 5:**
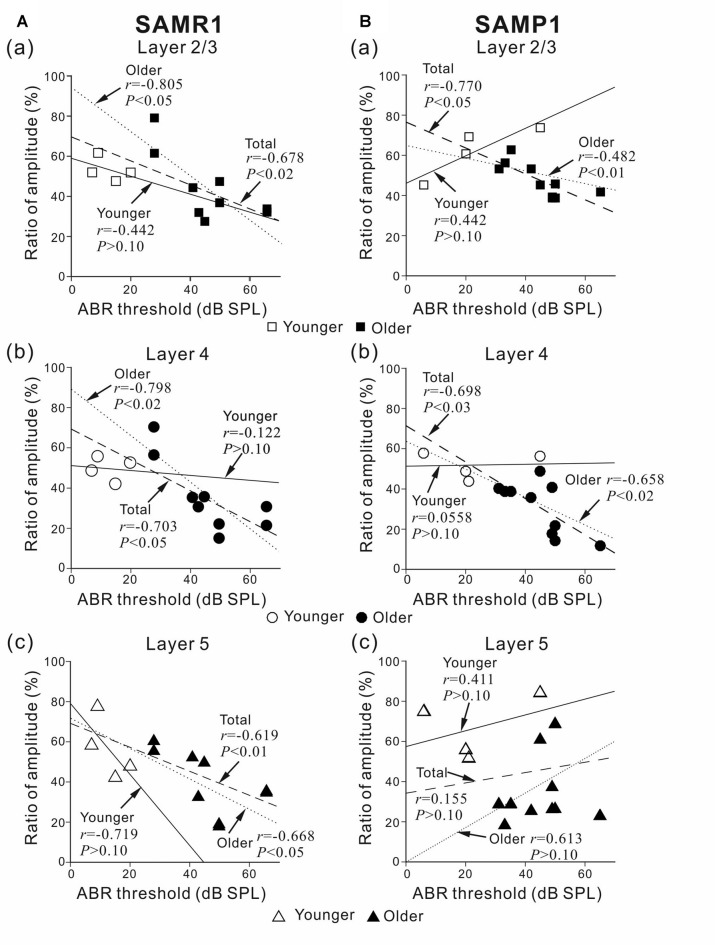
Plots showing the correlation between the ABR threshold and peak amplitude ratio before and after SS superfusion. **(A)** For younger and older SAMR1 mice, the correlation plots of L2/3, L4 and L5 are respectively shown in **(a–c)**. **(B)** Similarly, for younger and older SAMP1 mice, the correlation plots of L2/3, L4 and L5 are respectively shown in **(a–c)**. In each plot, *r* and *p*-values are written. Linear regression lines for the younger, older, and total (pooled) groups are plotted by straight, dotted, dashed lines, respectively. Data points for younger and older SAM are illustrated by open (□, ○ and △) and filled (■, ● and ▲) marks in the three layers (L2/3, L4 and L5), respectively.

**Figure 6:**
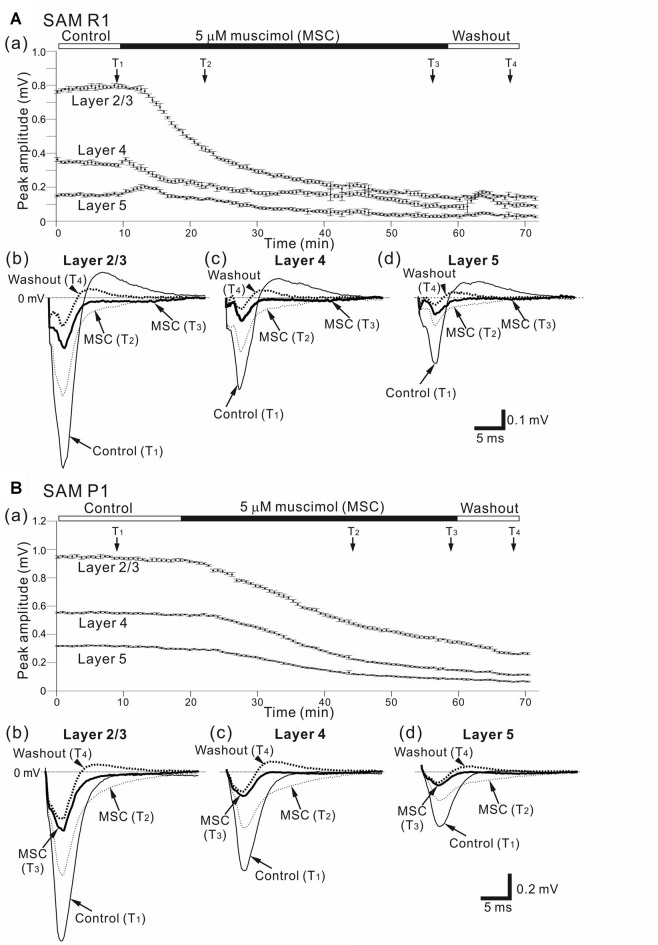
Effects of muscimol (MSC) on cortical LFP responses evoked by stimulation at one site in L4. **(A)** A representative example of an older SAMR1 mouse (12.5-month old). In **(a)**, superfusion of 5-μM MSC decreased the negative peak amplitudes of LFPs (T_2_ and T_3_) in the three layers (L2/3, L4 and L5), compared with control (T_1_). At T_4_, the drug had been washed out. Durations of the three (control, 5-μM MSC, and washout) conditions are given in the black and white bars on the top. Typical LFP responses in L2/3 **(b)**, L4 **(c)** and L5 **(d)** at the four time points (T_1_, T_2_, T_3_ and T_4_) are illustrated. **(B)** Similarly, a representative example of an older SAMP1 mouse (13-month old). In **(a)**, superfusion of 5-μM MSC also decreased the negative peak amplitudes of LFPs (T_2_ and T_3_), compared with control (T_1_). At T_4_, the drug had been washed out. Typical LFP responses in L2/3 **(b)**, L4 **(c)** and L5 **(d)** at the four time points (T_1_, T_2_, T_3_ and T_4_) are illustrated.

**Figure 7:**
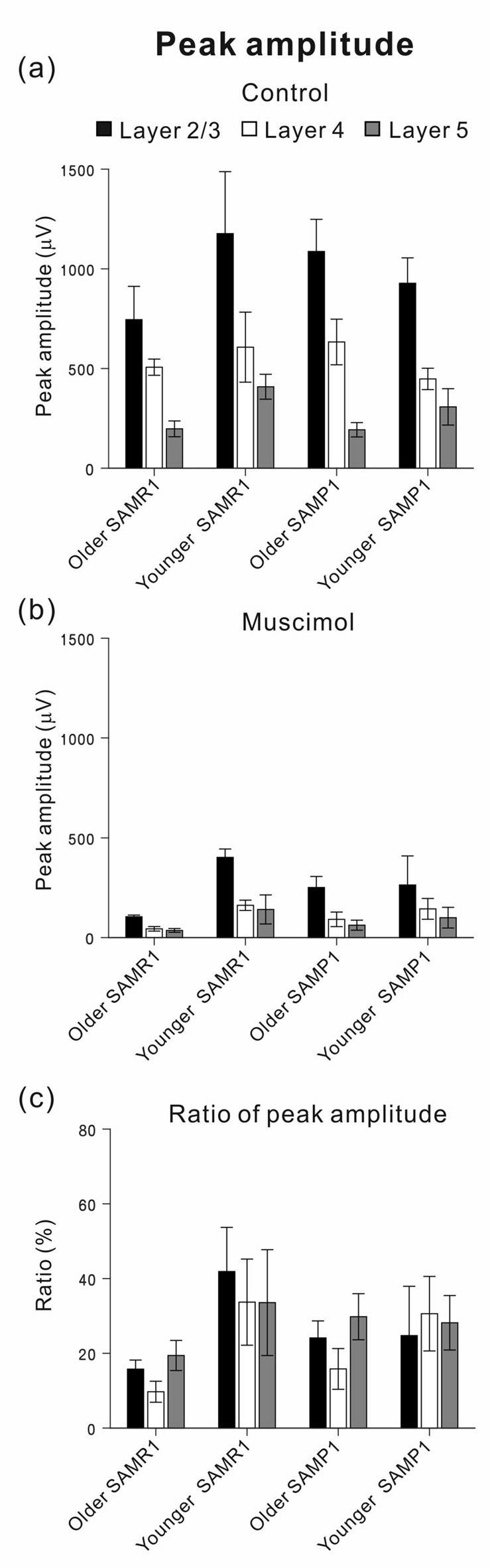
Among the four mouse groups (younger and older SAM-R1 and -P1), peak amplitudes of stimulus-driven LFP responses before and after MSC superfusion are shown. Peak amplitudes (*A*_ctl_ and *A*_MSC_) in L2/3 (black), L4 (white) and L5 (gray) among the mouse groups were plotted under the control condition in **(a)** and the 5-μM MSC superfusion condition in **(b)**, respectively. The ratios of the amplitudes (*A*_MSC_/*A*_ctl_) among the four mouse groups were also illustrated in **(c)**. Error bars represent SEM.

**Figure 8:**
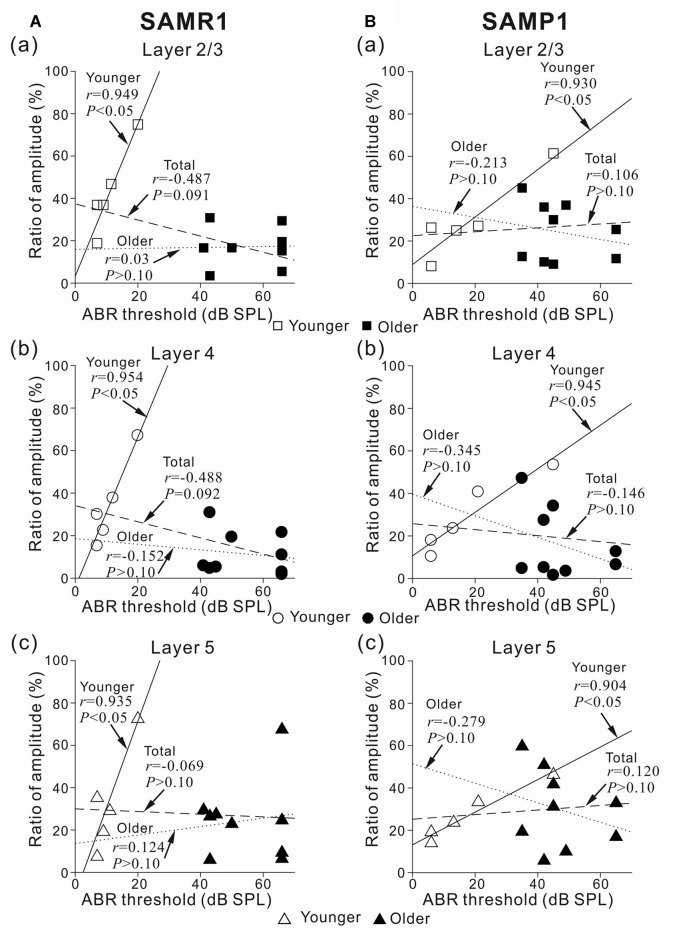
Plots showing the correlation between ABR threshold and peak amplitude ratio before and after the 5-μM MSC superfusion for younger and older SAMR1 and P1 mice. **(A)** For younger and older SAMR1 mice, the correlation plots of L2/3 (squares), L4 (circles), and L5 (triangles) are respectively shown in **(a–c)**. Data points for younger and older mice are illustrated by open (□, ○ and △) and filled (■, ● and ▲) marks in the three layers, respectively. Linear regression lines for the younger, older, and total (pooled) groups are plotted by straight, dotted, dashed lines, respectively. In each plot, *r* and *p*-values are reported. **(B)** Similarly, for SAMP1 mice, the correlation plots between ABR threshold and peak amplitude ratio for L2/3, L4 and L5 before and after the 5-μM MSC superfusion for younger and older SAMR1 are shown in **(a–c)**, respectively.

**Figure 9:**
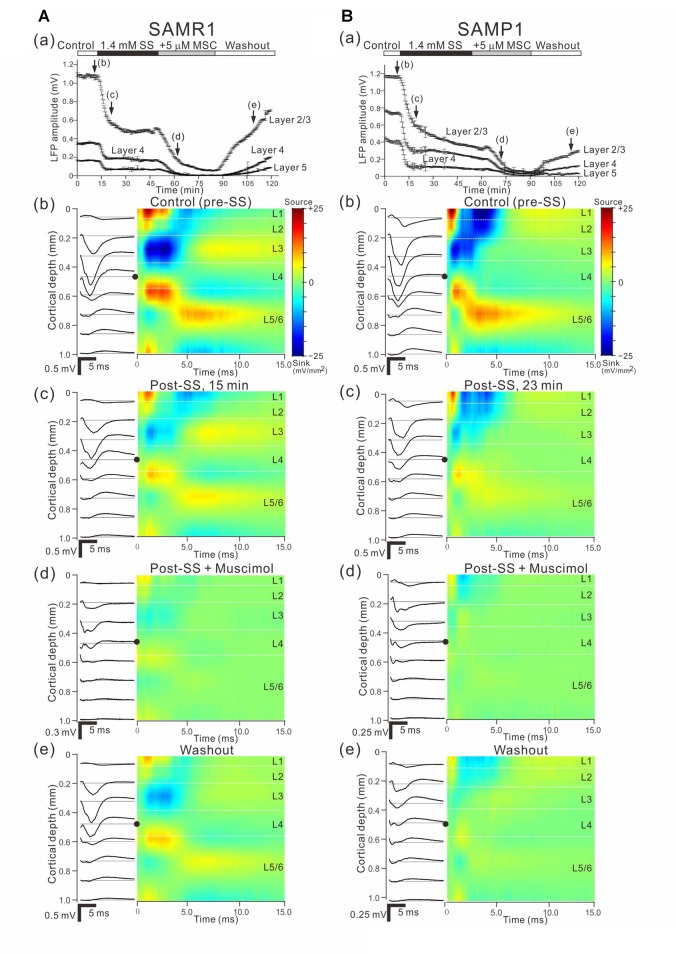
Effects of individual and simultaneous application of SS and MSC on cortical responses evoked by stimulation at one site in L4. **(A)** Representative example of an older SAMR1 mouse (13-month old). In **(a)**, following 1.4 mM SS administration (arrow **(c)**), superfusion of 5-μM MSC further decreased the negative peak amplitude of LFPs (arrow **(d)**) in the three layers (L2/3, L4 and L5), compared with control (arrow **(b)**). Durations of the four (control, post-SS, 5-μM MSC and washout) conditions are given in the white, back, gray and white bars on the top, respectively. **(b–e)** show the LFP (left) and current source density (CSD; right) profiles recorded at time points **(b–e)** in **(a)**, which respectively correspond to control (pre-SS), post-SS, post-SS plus MSC and washout. In the LFP profiles, the stimulation sites are indicated by black circles. All LFP and CSD values represent the averages over 10 trials. **(B)** Similarly, representative example of an older SAMP1 mouse (13-month old). As in Part **(A)**, the time courses of peak amplitudes in the three layers are shown in **(a)**, and the LFP (left) and CSD (right) profiles are illustrated under the four conditions in **(b–e)**.

**Figure 10:**
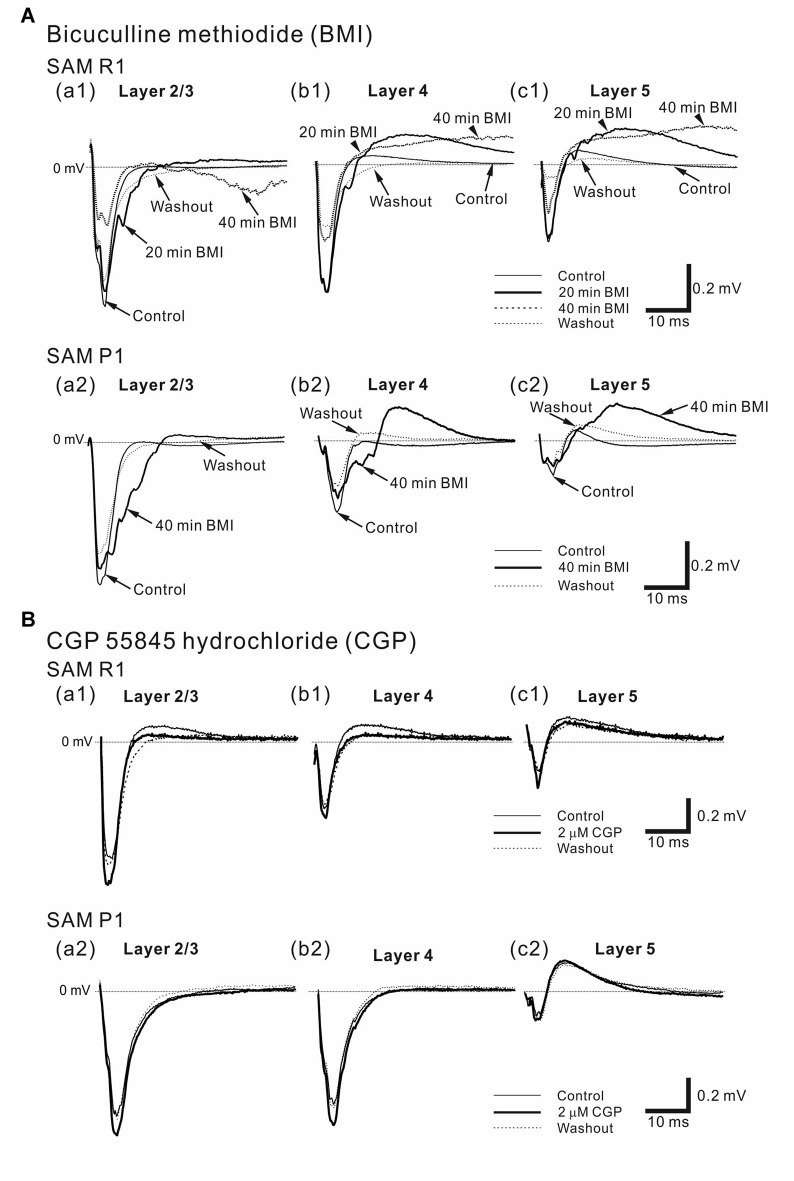
Effects of bicuculline methiodide (BMI) and CGP 55845 hydrochloride (CGP) on cortical LFP responses evoked by stimulation at one site in L4. **(A)** Typical examples of BMI effects on LFPs in older SAMR1 (13-month old) and SAMP1 (13-month old) mice. (top) In SAMR1, typical LFP responses in L2/3 **(a1)**, L4 **(b1)** and L5 **(c1)** are illustrated in the control, 4-μM BMI (20 and 40 min after superfusion), and washout conditions. (bottom) Similarly, in SAMP1, typical LFP responses in L2/3 **(a2)**, L4 **(b2)** and L5 **(c2)** are illustrated in the control, 4-μM BMI (40 min after superfusion), and washout conditions. **(B)** Representative examples of effects of CGP on LFPs in older SAMR1 (13-month old, top) and SAMP1 (13-month old, bottom) mice. (top) In SAMP1, typical LFP responses in L2/3 **(a1)**, L4 **(b1)** and L5 **(c1)** are illustrated in the control, 2-μM CGP (30 min after superfusion), and washout conditions. (bottom) Similarly, typical LFP responses in L2/3 **(a2)**, L4 **(b2)** and L5 **(c2)** are illustrated in the control, 2-μM CGP (30 min after superfusion), and washout conditions.

**Figure 11:**
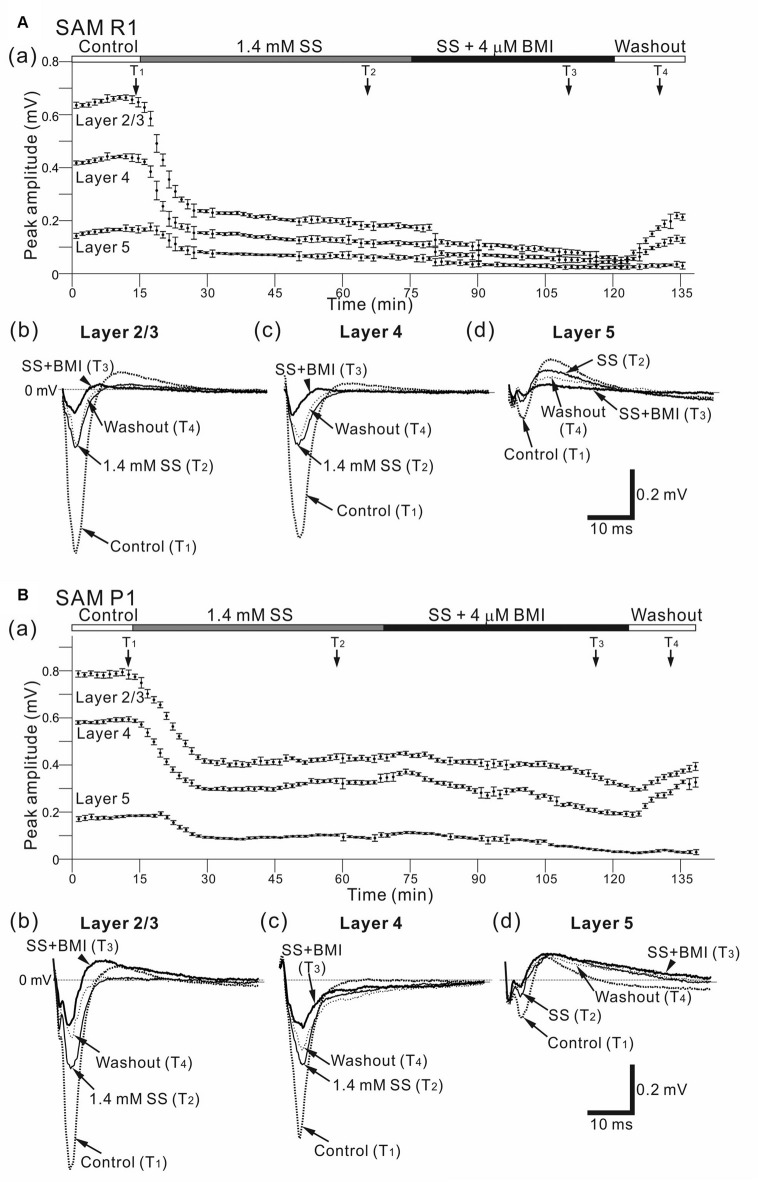
Effects of individual and simultaneous application of SS and BMI on cortical LFP responses evoked by stimulation at one site in L4. **(A)** Representative example of an older SAMR1 mouse (13-month old). In **(a)**, following 1.4 mM SS administration (T_2_), the superfusion of 4-μM BMI gradually decreased the negative peaks of LFP amplitudes (T_3_) in the three layers (L2/3, L4 and L5) compared with post-SS and control (T_1_). At T_4_ the drug had been washed out. Typical LFP responses in L2/3 **(b)**, L4 **(c)** and L5 **(d)** in each condition are illustrated. Durations of the four (control, post-SS, SS/BMI and washout) conditions are given in the white, gray, black and white bars on the top, respectively. **(B)** A representative example of an older SAMP1 mouse (13-month old). As in Part **(A)**, the time courses of peak amplitudes in the three layers are shown in **(a)**. The waveforms at the four time points (T_1_, T_2_, T_3_, and T_4_) in the three layers **(b–d)** are illustrated under the four conditions.

**Figure 12:**
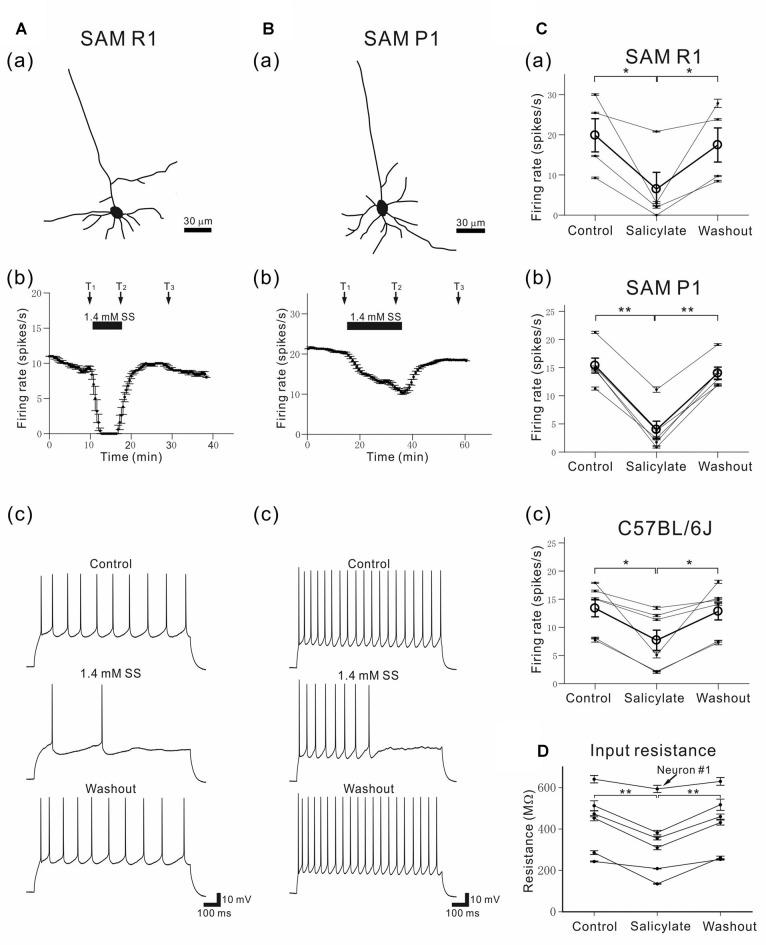
Morphology and characteristic firing of L4 pyramidal AC neurons in young SAM-R1 and -P1 mice. **(Aa)** A morphological trace of a typical pyramidal neuron from a SAMR1 mouse (see “Materials and Methods” section). **(b)** The time course of firing rates in the same pyramidal neuron after application of 1.4 mM SS. A horizontal bar indicates the duration of SS application. **(c)** Action potential trains evoked by a 1-s depolarizing current (150 pA) in a pyramidal neuron before (top panel; at time T_1_ in the control condition), during (middle panel; at time T_2_ in the 1.4-mM SS condition), and after (bottom panel; at time T_3_ in the washout condition) application of SS. **(Ba)** A morphological trace of a typical pyramidal neuron from a SAMP1 mouse.**(b)** The time course of firing rates in a pyramidal neuron afterapplication of 1.4 mM SS. Vertical bars represent SEM.**(c)** Action potential trains evoked by a 1-s depolarizing current (150 pA). **(C)** Changes in action potential trains evoked by a 1-s depolarizing current (100–150 pA) in a pyramidal neuron before (control), during (1.4-mM SS) and after (washout) application of SS for the three mouse strains: SAMR1 in **(a)**, SAMP1 in **(b)** and C57BL/6J in **(c)**. For group data, the mean firing rate is represented by open circles and thick lines. Vertical bars represent SEM. *P < 0.05 and **P < 0.01. **(D)** Membrane input resistance of L4 pyramidal neurons in C57BL/6J mice before (control), during (salicylate) and after (washout) the superfusion of 1.4 mM SS. The input resistance of one neuron (neuron #1) out of six neurons was not significantly different among the three conditions (*P* > 0.10), and that of other five neurons was significantly different (**P < 0.01) by one way analysis of variance (ANOVA) with the Tukey-Kramer multiple comparison.

### Drug Application

After stable baseline recordings had been acquired for longer than 10 min, we administered SS (1.4 mM, S3007, Sigma-Aldrich, St. Louis, MO, USA), the GABA_A_ receptor agonist muscimol (MSC; 5 μM, Cat. No., 0289, Tocris, UK), the GABA_A_ receptor antagonist bicuculline methiodide (BMI; 4 μM, Cat. No., 2503, Tocris), and the GABA_B_ receptor antagonist CGP 55845 hydrochloride (CGP; 2 μM, Cat. No. 1248, Tocris) for 10–30 min (e.g., Figures 3Aa,Ba). We used an SS concentration of 1.4 mM, as this is the concentration found in the cerebrospinal fluid of tinnitus animal models developed with SS (Deer and Hunter-Duvar, 1982; Jastreboff et al., 1986). This concentration also facilitated comparison of our results with those of previous studies (Wang et al., 2006; Su et al., 2009).

We administered SS and a receptor agonist/antagonist individually or in combination. Under each drug condition, we examined the properties of stimulus-driven LFPs and action potentials when a drug-induced increase/reduction reached a stable level with no further changes over a period of at least 5 min. In each condition, SS was the first drug released. In the combination condition, we first waited until the effect of SS had reached a stable level, and then released a neurotransmitter receptor agonist/antagonist while maintaining application of SS. We compared LFP changes caused by simultaneous application of SS and a receptor agonist/antagonist with changes caused by application of each drug individually. We expected a disparity between the effects of simultaneous vs. individual drug application, especially for MSC-induced-effects on evoked LFPs. We interpreted such interactions to suggest that SS modulated GABAergic pathways/neurotransmission.

In all LFP recordings, recovery was usually observed until stimulus-driven LFPs were reduced to a voltage level below 50 μV. However, in some drug applications, we observed no recovery of LFPs in the washout condition. We evaluated the effect of a drug/drugs on stimulus-driven LFPs by comparing the peak amplitude, latency and half width obtained before and during application of the drug/drugs. Similarly, action potential properties including amplitude, half width and firing rate were examined before and after administering SS.

### Statistical Data Analysis

We analyzed data from 71 mice: the MEA recording experiments involved 79 AC slices from 51 animals (20 SAMP1, 18 SAMR1, and 13 C57BL/6J mice, Table 1) and the whole-cell patch-clamp recording experiments included 21 AC slices from 20 animals (four SAMP1, five SAMR1, 11 C57BL/6J mice). Data are expressed as means ± the standard error of the mean (SEM). Error bars represent SEM. A value of *P* < 0.05 was considered to be significant. Statistical comparisons involving two groups were performed using the Student’s *t*-test, and comparisons for multiple groups were performed using one-way or two-way analysis of variance (ANOVA) for multiple comparisons followed by the Tukey-Kramer test as a *post hoc* test (Matlab 2015a, MathWorks, Natick, MA, USA). Correlations between two variables were calculated using Pearson’s correlation coefficients.

## Results

### ABR Properties in SAMR1, SAMP1 and C57BL/6J Mice

First, we describe the ABR properties obtained from the three mouse strains: SAMR1, SAMP1 and C57BL/6J. As reported previously (Saitoh et al., 1994), typical *in vivo* ABRs from the SAM as well as C57BL/6J mice featured a series of five prominent positive peaks (wave I to V) in response to a short click sound (Figure 1A). ABR thresholds were not significantly different among the younger groups (group A) from each of the three mouse strains (Table 1; 13.3 ± 1.8 dB SPL in SAMR1, *n* = 9; 19.8 ± 1.6 dB SPL in SAMP1, *n* = 9; 23.3 ± 7.2 dB SPL in C57BL/6J, *n* = 13; *P* > 0.05 by ANOVA with the Tukey-Kramer multiple comparison test). Similarly, ABR thresholds were not significantly different among the older SAMR1 and SAMP1 groups (group B; 47.4 ± 4.9 dB SPL in SAMR1, *n* = 12; 44.4 ± 3.6 dB SPL in SAMP1, *n* = 11; *P* > 0.10 by *t*-test). In short, we found that the ABR thresholds of the two SAM strains were significantly different only when we compared the younger vs. older groups (*P* < 0.05; Figure 1B).

Quantification of the local peak amplitudes of the obtained suprathreshold ABRs to a 65 dB SPL click revealed a significant difference in wave I amplitude among the total group data for the younger SAM mice (*P* < 0.05; Figure 1Ca). However, we observed no significant amplitude differences among the rest of the waves (waves II to V) in the younger SAM groups, although we observed some variety in amplitudes in individual data (Figure 1Ca). In the older SAM groups, we only observed significant amplitude differences in wave III (*P* < 0.05; Figure 1Ca). We found no significant differences in latency for any waves among all age and strain groups (Figure 1Cb).

With regard to the comparison between younger vs. older SAM strains, in the SAMR1 strain waves I, II and IV, but not wave III and V in ABR recording showed significant age-dependent decreases in their amplitude (Figure 1Ca). On the other hand, for the SAMP1 strain, significant age-dependent decreases in amplitude only occurred in waves II and III (Figure 1Ca). The results indicated that in both strains, an age-dependent loss of hearing can be observed for some of the five waves in a matter that depended on the strains. However, more server hearing loss expected in SAMP1 than SAMR1 was not observed in our experiments.

### Spatiotemporal LFP Activity Evoked in L4 in Slices from Younger SAMR1 and SAMP1 Mice

To examine the strain differences in evoked responses to short current stimulation in younger mice, we recorded cortical LFPs from AC slices. For all AC slices (Figure 2A), stimulation at single sites in L4 (Figure 2B), which is a main recipient layer from neurons in the ventral division of the medial geniculate body (vMGB), evoked large negative LFP responses with short latencies in L2/3 (Figure 2C), L4, and layer 5 (L5; c.f., Figure 2B). The peak amplitudes of LFP responses in L4 of the younger mice were not significantly different among the three strains: 0.803 ± 0.151 mV in SAMR1 (*n* = 7 slices), 0.790 ± 0.123 mV in SAMP1 (*n* = 7), and 0.810 ± 0.090 mV in C57BL/6J (*n* = 12; *P* > 0.10 by Tukey-Kramer multiple comparison test). Additionally, the corresponding peak latencies were not significantly different: 3.41 ± 0.12 ms in SAMR1, 3.84 ± 0.17 ms in SAMP1 and 3.47 ± 0.11 ms in C57BL/6J (*P* > 0.10). Further, in the younger mice, half widths were not significantly different: 3.68 ± 0.18 ms in SAMR1, 3.89 ± 0.19 ms in SAMP1 and 3.59 ± 0.09 ms in C57BL/6J (*P* > 0.10). Thus, our results indicate that stimulation of L4 elicited no significant differences in evoked LFP responses in the stimulation layer (L4) in younger mice from the three mouse strains. These results were consistent with those reported for SAM and C57BL/6J mice in our previous study (Yamamura et al., 2017).

### SS-Induced Effects on LFPs in AC Layers from SAM Mice

Before and after administering 1.4 mM SS, we examined cortical layer-specific changes in LFPs among the SAM strains. For AC slices from younger and older SAMR1 and SAMP1 mice, we first recorded LFPs in L2/3, L4 and L5 in the control (pre-SS) condition for 10 min (Figures 3Aa,Ba). Then, we administered SS to brain slice preparations via superfusion of an ACSF solution with SS. The drug was subsequently washed out. In a typical slice from an older SAMR1 mouse, the peak amplitude during the early negative-going component of LFPs in L2/3, L4 and L5 gradually decreased after the 1.4-mM SS superfusion (Figure 3Aa), and SS application significantly reduced the peak amplitude (*A*_SS_) in L2/3 to around 30% of the initial amplitude (*A*_ctl_) (indicated by an arrow with T_2_ in Figure 3Aa; *A*_ctl_ = 1.22 ± 0.05 mV vs. *A*_SS_ = 0.457 ± 0.08 mV, *P* < 0.01 by *t*-test). All amplitude change data for SAMR1 mice are summarized in Figure 4. About 20 min after the offset of SS administration (e.g., T_3_ in Figure 3Aa), the LFP amplitudes in all layers approached the pre-drug-release levels or were slightly smaller. This indicates that the effect of 1.4-mM SS was reversible.

In the three conditions (pre-SS, post-SS and washout), the LFP waveforms at three time points (T_1_, T_2_ and T_3_ in Figure 3Aa) in the older SAMR1 mice (Figures 3Ab–d) showed layer-specific changes. Particularly, in L2/3 (Figure 3Ab), the amplitude of the negative LFP peak significantly decreased after SS perfusion. The decay time constant (τ_d_) of the amplitude change in L2/3 was 10.4 ± 1.0 min. Similarly, in L4 and L5, the negative peak amplitudes significantly decreased after SS perfusion (Figures 3Ac,d). In L4 and L5, the time constants τ_d_ were 9.9 ± 1.5 and 11.5 ± 1.2 min, respectively, and they were not significantly different among all layers (*P* > 0.10). About 20 min after cessation of SS administration, LFP waveforms were restored to pre-drug-release shapes in all layers, indicating that the effect of the 1.4-mM SS application on LFP waveforms was reversible (Figures 3Ab–d). The group data are summarized in Figures 4a,b. However, the peak latencies and half widths in the SAMR1 strain were not significantly altered in any layer (see Table 2 and Supplementary Figure S1). Additionally, we found no significant differences in amplitude ratio (*A*_SS_/*A*_ctl_) among the three layers (L2/3, L4 and L5) between the control vs. SS condition in older or younger SAMR1 mice (Figure 4c, *P* > 0.05 by Tukey-Kramer multiple comparison test). Thus, we observed no clear SS-induced differences between the three layers in the SAMR1 strain.

**Table 2:** Layer-specific response properties of local field potentials (LFPs) driven at one site in L4 of younger and old senescence-accelerated mice (SAM) mice.

Mouse strain	Age group	Layer	Peak latency in ms		Half width in ms	
			Control	Sodium salicylate	Control	Sodium salicylate
SAMR1	Younger	2/3	3.80 ± 0.23	3.86 ± 0.18	3.94 ± 0.42	3.16 ± 0.36
		4	3.41 ± 0.12	3.78 ± 0.08	3.48 ± 0.18	3.36 ± 0.34
		5	3.66 ± 0.11	3.99 ± 0.20	3.00 ± 0.16	2.90 ± 0.33
	Older	2/3	3.83 ± 0.18	3.47 ± 0.22	4.41 ± 0.19	4.34 ± 0.46
		4	3.53 ± 0.16	3.51 ± 0.10	3.68 ± 0.26	3.43 ± 0.44
		5	3.64 ± 0.42	3.74 ± 0.41	2.71 ± 0.38	2.44 ± 0.47
SAMP1	Younger	2/3	4.06 ± 0.11	4.09 ± 0.08	4.20 ± 0.18	4.01 ± 0.13
		4	3.84 ± 0.17	3.49 ± 0.07	4.01 ± 0.17	3.98 ± 0.15
		5	3.92 ± 0.14	3.69 ± 0.08	3.59 ± 0.20	3.48 ± 0.22
	Older	2/3	3.84 ± 0.22	3.94 ± 0.27	3.94 ± 0.17	4.07 ± 0.28
		4	3.67 ± 0.18	3.49 ± 0.18	3.47 ± 0.17	3.19 ± 0.22
		5	3.98 ± 0.25	3.83 ± 0.11	3.32 ± 0.16	2.87 ± 0.16

*Data are shown as the mean ± SEM*.

Figure 3Ba shows the peak amplitude during the early negative-going LFP component in the three layers in a typical slice from an older SAMP1 mouse. After SS superfusion, the LFP amplitude in L2/3 was significantly reduced (*A*_ctl_ = 1.15 ± 0.04 mV vs. *A*_SS_ = 0.487 ± 0.03 mV, *P* < 0.01 by *t*-test) and reached a stable level (T_2_ in Figure 3Ba). In L2/3, L4 and L5, the decay time constants τ_d_ were 16.9 ± 1.2, 17.1 ± 1.6 and 16.2 ± 1.5 min, respectively, and they were not significantly different among all layers. In addition, the time constants in the older SAMP1 mice were significantly different from those in the older SAMR1 (*P* < 0.05 by ANOVA with Tukey-Kramer multiple comparison test). Thus, the time courses of amplitude reduction in the older SAMP1 mice were slower than those in the older SAMR1 mice. About 25 min after SS administration offset, the peak amplitude generally returned to the original level (T_3_ in Figure 3Ba), indicating that the effect of 1.4-mM SS application on peak amplitude was also reversible in SAMP1 mice (Figures 3Bb–d). Unlike the SAMR1 mice, the LFP waveforms in all layers for older SAMP1 mice showed similar changes in the three conditions (Figures 3Ab–d). Figure 4 summarizes all data regarding amplitude changes in all layers in SAMP1 mice. In L2/3 of the older SAMP1 mice, we did not observe a clear positive peak following the negative peak in each LFP (*n* = 7 out of nine slices from six animals, Figure 3Bb). About 25 min after SS administration offset (T_3_ in Figure 3Ba), the LFP waveforms in all layers assumed their pre-drug-release shapes, although the LFP waveforms in L4 and L5 showed a post-drug-release effect in that the half width of the LFPs increased in some slices (*n* = 5 out of nine slices, Figures 3Bc,d). The same effect was observed in the other two layers (Figures 4a,b). Before and after SS superfusion, the peak latencies and half widths did not change significantly in any layers in SAMR1 mice (see Table 2).

In summary, among the three layers in the older SAMP1 group, the amplitude ratios (*A*_SS_/*A*_ctl_) in L4 and L5 were significantly smaller than those in L2/3 (*P* < 0.05 by Tukey-Kramer multiple comparison test; Figure 4c), indicating that SS-induced effects were more prominent in L4 and L5 than in L2/3 in older SAMP1 mice. In contrast, we found no significant differences in the amplitude ratio among the three layers (*P* > 0.10 by Tukey-Kramer multiple comparison test, Figure 4c) in the other mouse groups (younger and older SAMR1 and younger SAMP1 mice).

To investigate whether the SS-induced reduction in LFP peak amplitude was associated with the sensitivity of the auditory periphery in the tested mice, we calculated correlation coefficients (*r*-values) between ABR thresholds and the ratio of peak amplitudes before and after the SS superfusion in individual younger and older group data and the total (pooled) data (Figure 5). As a result, we found strong negative correlations in L2/3 and L4 in SAMR1 mice of the total groups (*r* = −0.678, *P* < 0.02 in L2/3; *r* = −0.703, *P* < 0.05 in L4; Figures 5Aa,b) and those of the older group (*r* = −0.805, *P* < 0.05 in L2/3; *r* = −0.798, *P* < 0.02 in L4) and a moderately strong correlation in L5 of the total groups (*r* = −0.619, *P* < 0.01, Figure 7) and the older group (*r* = −0.668, *P* < 0.05). Thus, in SAMR1 mice with hearing loss, the SS-induced reduction of LFPs was profoundly larger in all examined layers, and the SS effect in L5 tended to be slightly weaker than that in L2/3 and L4. Similarly, in SAMP1 mice, we found a strong negative correlation between ABR thresholds and amplitude ratios in L2/3 and L4 of the total groups (*r* = −0.770, *P* < 0.01 in L2/3; *r* = −0.698, *P* < 0.03 in L4; Figures 5Ba,b) and the older group (*r* = −0.482, *P* < 0.01 in L2/3; *r* = −0.658, *P* < 0.02 in L4). In contrast, in L5 of SAMP1 mice, we found no such tendencies in the two groups (Figure 5Bc). Thus, we found a difference between the two SAM strains in terms of cortical layer-specific SS-induced effects on stimulus-driven LFPs. Additionally, in the two SAM strains with a greater degree of hearing loss (ABR threshold ≥45 dB SPL), the SS-induced reduction of LFPs was significantly larger in L2/3 and L4 compared with L5 (*P* < 0.05; Figures 5A,B).

### GABAR-Agonist Effects on LFPs in AC Layers from SAM Mice

Here, we examined how a GABA_A_ receptor agonist, MSC, influenced stimulus-driven LFPs in a layer-dependent manner (Figure 6). For AC slices from younger and older SAMR1 and SAMP1 mice, we first recorded LFPs in L2/3, L4 and L5 in the control (pre-MSC) condition for 10 min (Figures 6Aa,Ba). Subsequently, we administered 5 μM MSC to the slice preparations via superfusion of an ACSF solution with MSC, and then washed out the drug after reaching a stable level of amplitude changes in LFPs.

Figure 6Aa shows a typical time course of LFP amplitudes in an older SAMR1 mouse. The negative peak amplitudes of stimulus-driven LFPs in L2/3, L4 and L5 gradually decreased after MSC superfusion for the initial amplitude (*A*_ctl_). The MSC application significantly reduced the peak amplitude (*A*_MSC_) in L2/3 to around 14.1% (100 × *A*_MSC_/*A*_ctl_) of the initial amplitude (indicated by an arrow with T_3_ in Figure 6Aa; *A*_ctl_ = 0.745 ± 0.167 mV vs. *A*_MSC_ = 0.105 ± 0.08 mV, *n* = 8; *P* < 0.01 by *t*-test). In L2/3, L4 and L5, the decay time constants τ_d_ were 13.1 ± 1.4, 12.8 ± 1.1 and 13.7 ± 1.1 min, respectively, and they were not significantly different among all layers (*P* > 0.10). LFP amplitudes in all layers had not returned to pre-drug-release levels 10 min after offset of MSC administration (e.g., T_4_ in Figure 6Aa) or at any point during continuous recording. Thus, although we observed slight increases in the positive peaks in the washout condition for the SAMR1 mice in three slices out of eight, the LFP amplitudes did not return to pre-drug-release levels in any slices. Figure 7 summarizes the data regarding changes in negative peak amplitudes in SAMR1 mice.

In the three conditions (pre-MSC, post-MSC and washout) for the older SAMR1 mice (Figures 6Ab–d), the LFP waveforms exhibited layer-independent changes at four time points (T_1_, T_2_, T_3_ and T_4_ in Figure 6Aa). In all layers, MSC superfusion led to a significant reduction in the amplitudes of negative LFP peaks, as described above. In addition, the positive LFP peaks following the negative peaks (see control LFP waveforms at T_1_ in Figures 6Ab–d) gradually decreased and finally disappeared more than 10 min after MSC application (e.g., at time points T_2_ and T_3_ in Figures 6Ab–d). Moreover, about 10 min after MSC administration offset (during the washout condition), the LFP waveforms exhibited small positive LFP peaks (LFP waveforms at T_4_ in Figures 6Ab–d) in all layers, although these waveforms did not assume their pre-drug-release shapes. These results indicate that the negative peaks were associated with GABA_A_-receptor mediated components in the LFPs, which is consistent with a previous study (Connors et al., 1988). Also, after 20 min, and longer during the washout condition, the LFP waveforms were not the same as the control waveforms.

With respect to additional characteristic properties of stimulus-driven responses in older SAMR1 mice, before and after SS application, the peak latencies and half widths of the LFPs in the SAMR1 strain did not significantly change in any layer (see Supplementary Figure S2). Further, among the three layers (L2/3, L4 and L5), we found no significant differences in amplitude ratio (*A*_MSC_/*A*_ctl_) between the control and MSC conditions in both older and younger SAMR1 mice (Figures 7a,c, *P* > 0.10 by Tukey-Kramer multiple comparison test). Thus, we found no clear MSC-induced differences between the three layers in the SAMR1 mice.

Figure 6Ba shows a typical LFP amplitude time course in an older SAMP1 mouse. After MSC superfusion, the amplitude in L2/3 was significantly reduced (*A*_ctl_ = 0.997 ± 0.161 mV at T_1_ vs. *A*_MSC_ = 0.251 ± 0.055 mV at T_3_, *P* < 0.01 by *t*-test). The MSC application significantly reduced the peak amplitude (*A*_MSC_) in L2/3 to around 25.2% (100 × *A*_MSC_/*A*_ctl_) of the initial amplitude. In L2/3, L4 and L5, the decay time constants τ_d_ were 19.8 ± 1.6, 19.3 ± 1.9, and 20.3 ± 1.5 min, respectively, and they were not significantly different among all layers (*P* > 0.10). Thus, the time courses of amplitude reduction in the older SAMP1 mice seemed to be slower than those in the older SAMR1 mice (Figure 6Ba; see, Figure 6Aa), although the time constants in the older SAMP1 mice were not significantly different from those in the older SAMR1 (*P* > 0.05 by Tukey-Kramer multiple comparison test). Moreover, more than 10 min after MSC administration offset (during washout) and during continuous recording, the LFP waveforms in all layers exhibited small positive LFP peaks (LFP waveforms at T_4_ in Figures 6Bb–d), although the LFPs did not assume their pre-drug-release shapes. Figure 7 summarizes all the group data regarding amplitude changes in examined SAMP1 mice.

For the older SAMP1 mice, the LFP waveforms exhibited similar changes in the three conditions (pre-MSC, post-MSC, and washout) among the three layers (Figures 6Bb–d). This result was consistent with that obtained for the older SAMR1 mice (Figures 6Ab–d). However, in the control condition, we did not observe a clear positive peak following the negative peak in each LFP in the older SAMP1 (*n* = 7 out of 9 slices from different animals). This result deviated from that in the older SAMR1 mice. Therefore, administration of MSC caused no clear reduction in the positive LFP peaks. Over 10 min after the cessation of MSC administration (T_4_ in Figure 6Ba), we unexpectedly found a small positive peak following the negative peak in each LFP waveform for all layers (washout waveforms at T_4_ in all layers; *n* = 6 out of 9 slices from six animals).

Before and after MSC superfusion, the peak latencies and half widths of the LFPs from the older SAMP1 mice did not significantly change in any layer (see Supplementary Figure S3). Among the three layers in the older SAMP1 group, we found no significant differences in the amplitude ratio (*A*_SS_/*A*_ctl_) (*P* > 0.10 by Tukey-Kramer multiple comparison test; Figure 7c), indicating that, like in older SAMR1 mice, the MSC-induced effects were not significantly different in any layers in older SAMP1 mice.

To investigate whether the MSC-induced reduction in LFP peak amplitude was associated with the sensitivity of the auditory periphery in the tested SAM, we calculated correlation coefficients (*r*-values) between ABR thresholds and the ratio of peak amplitudes in all layers before and after MSC superfusion (Figure 8). The results indicated strong positive correlations in all layers in the younger SAMR1 (*r* = 0.949, *P* < 0.05 in L2/3; *r* = 0.954, *P* < 0.05 in L4; *r* = 0.935, *P* < 0.05 in L5; Figure 8A) and younger SAMP1 (*r* = 0.930, *P* < 0.05 in L2/3; *r* = 0.946, *P* < 0.05 in L4; *r* = 0.904, *P* < 0.05 in L5; Figure 8B). However, for the total data of the younger and older groups in both older SAM strains, the relationships between ABR thresholds and the ratio of peak amplitudes in all layers showed no clear correlations among data points (Figure 8). The tendency of no clear correlation was similar to the older group data in the both strains. Therefore, we found no cortical layer-specific differences in MSC-induced effects on the amplitudes of stimulus-driven LFPs between the two SAM strains.

### Effects of Simultaneous Administration of SS and MSC on Evoked LFPs and CSDs

Next, we examined how stimulus-driven LFP responses were affected by the combined application of SS and MSC. As described in the previous subsections, SS and MSC consistently reduced LFP amplitudes when individually administered to both older SAM-R1 and -P1 mice. In older SAMR1 mice, as described above, SS application reduced the peak amplitude of evoked LFPs in all layers; in L2/3, *A*_ctl_ = 0.882 ± 0.227 mV vs. *A*_SS_ = 0.268 ± 0.073 mV, *n* = 7, *P* < 0.01 by *t*-test, which corresponds to the reduction of the peak amplitude to 30.4% (100 × A_SS_/A_ctl_) from the control condition. Further, the addition of MSC after administration of SS suppressed the peak amplitudes (*A*_SS + MSC_) of evoked LFPs in all layers (L2/3, L4, and L5; Figure 9Aa); in L2/3, *A*_SS_ = 0.268 ± 0.073 mV vs. *A*_SS + MSC_ = 0.057 ± 0.008 mV, *n* = 7, *P* < 0.01 by *t*-test, which corresponds to the reduction of the LFP peak amplitude to 21.3% (100 × A_SS + MSC_/A_SS_) of the amplitude by the MSC application in the presence of SS. The total decrease in LPF waveforms was larger under the simultaneous application condition (left column of Figure 9Ad) compared with administration of SS only (left column of Figure 9Ac). We observed a similar tendency in older SAMP1 mice. SS application reduced the peak amplitude of evoked LFPs in all layers; in L2/3, *A*_ctl_ = 1.249 ± 0.251 mV vs. *A*_SS_ = 0.431 ± 0.145 mV, *n* = 7, *P* < 0.01 by *t*-test, which corresponds to the reduction of the peak amplitude to 34.5% from the control condition. The addition of MSC further reduced the LFP amplitude, compared with the initial SS treatment; *A*_SS_ = 0.431 ± 0.145 mV vs. *A*_SS + MSC_ = 0.127 ± 0.036 mV, *n* = 7, *P* < 0.01 by *t*-test, which corresponds to the reduction of the LFP peak amplitude to 29.5% of the amplitude by the MSC application with the presence of SS. Moreover, in the SAM strains, we examined the amount of LFP peak amplitude decreased by MSC in the presence and absence of SS. To examine the interaction between SS and MSC on LFP peak amplitude for the strain group data in all layers, we compared the two amplitude ratios A_SS + MSC_/A_SS_ (Figure 9) and A_MSC_/A_ctl_ (Figure 6). The result showed that the differences of the ratios in all layers were not statistically significant in the two strains (two-way ANOVA followed by the Tukey-Kramer test), suggesting that no interactions of SS and MSC and that SS administration did not influence the GABAergic receptor-mediated changes in the evoked LFPs through MSC.

With regard to the stimulus-driven LFP responses, we analyzed the corresponding CSD profiles (Figures 9A,Bb–e). After stimulation onset in L4 of SAMR1 mice, we identified source–sink–source triplet CSD profiles from L1 to L5 during a period from 1 ms to 3 ms after stimulus onset (hereafter the “earlier phase”; Figure 9Ab). In response to acoustic sound stimulation, the triplet profiles were reminiscent of those observed in *in vivo* recordings of the A1 in mice (Cruikshank et al., 2002) and rats (Sakata and Harris, 2009). In the triplet, the first transition from source to sink occurred in L2 or the border between L2 and L3, while the second transition from sink to source occurred in L4 or the border between L4 and L5 (Yamamura et al., 2017). After the triplet appeared in the CSD patterns, we observed a pair of small sink and source events in L4 and L5/6 over a period of 3–4 ms (hereafter the “later phase”) after stimulus onset (Figure 9Ab). After administering SS, the peaks of the sources and sinks in the earlier phase gradually decreased, although the triplet patterns in the earlier phase and sink-source patterns in the later phase remained (Figure 9Ac). In terms of the CSD profiles during simultaneous application of SS and MSC, the triplet patterns in the earlier phase remained while the sink-source patterns in the later phase were almost completely absent. After the washout, the sink-source patterns in the CSD profiles were partially recovered. Moreover, in SAMP1 mice, CSD profiles in the early phase were similar to those seen in SAMR1 mice, although the sinks in the later phase were not clearly detected (four slices out of five). Following SS-administered sink-source reduction (Figure 9Bc) in the SAMP1 mice, application of MSC further decreased the peaks in the CSD profiles (Figure 9Bd). After the washout, the triplets in the early phase were partially recovered, although the sink-source patterns in the later phase were not clearly detected (Figure 9Be).

### Effects of GABA_A_ and GABA_B_ Antagonists on Evoked LFPs

To examine the disinhibitory effects of a GABA_A_ receptor antagonist (BMI) on local AC networks in SAM mice, we recorded stimulus-driven LFPs during 4 μM BMI superfusion (Figure 10A). Approximately 40 min after administering BMI, the negative peak amplitude (*A*_BMI_) of the LFP response in older SAMR1 mice (Figures 10Aa1–c1) had significantly decreased compared with the amplitude (*A*_ctl_) in the pre-BMI control condition (*A*_BMI_/*A*_ctl_ = 0.56 ± 0.16 in L2/3, 0.71 ± 0.10 in L4, 0.64 ± 0.14 in L5; *n* = 6; *P* < 0.05). Subsequently, in the washout condition, the LFP waveforms were not fully recovered to those in the pre-BMI condition (Figures 10Aa1–c1).

In contrast, in older SAMP1 mice (Figures 10Aa2–c2), the negative peak amplitude of the LFP response had not significantly changed 40 min after administering BMI (*A*_BMI_/*A*_ctl_ = 0.86 ± 0.11 in L2/3, 0.85 ± 0.13 in L4, 0.81 ± 0.12 in L5; *n* = 5; *P* > 0.10), although we observed weak amplitude reduction tendencies in all layers (Figures 10Aa2–c2).

For AC slices in younger SAM-R1 and -P1 mice, the response amplitudes of stimulus-driven LFPs did not significantly change within the first 20 min after BMI administration (for SAMR1, *A*_BMI_/*A*_ctl_ = 1.06 ± 0.14 in L2/3, 1.02 ± 0.10 in L4, 0.96 ± 0.10 in L5; *n* = 6; *P* > 0.10; for SAMP1, *A*_BMI_/*A*_ctl_ = 0.85 ± 0.13 in L2/3, 0.83 ± 0.12 in L4, 0.81 ± 0.18 in L5; *n* = 4; *P* > 0.10). After 20 min, the LFP responses largely oscillated (data not shown here). Finally, around 40 min after BMI application, the response amplitudes abruptly reduced to nearly zero for all tested slice preparations. Once the LFP responses had diminished, we obtained no further responses. Therefore, the effects of BMI on LFP responses in younger SAM mice were profoundly different from those obtained in older SAM mice under the same conditions.

We next examined the stimulus-driven LFP responses produced by simultaneous administration of multiple GABA_A_ receptor antagonists after SS (Figure 11). Following SS administration, simultaneous application of two drugs (SS and BMI) led to a significant decrease (compared with amplitudes in the SS alone condition) in negative peak LFP amplitudes (*A*_SS + BMI_) in older SAMR1 and SAMP1 mice for all layers (for SAMR1, *A*_SS + BMI_/*A*_SS_ = 0.82 ± 0.05 in L2/3, 0.79 ± 0.11 in L4, and 0.81 ± 0.06 in L5; *n* = 4; for SAMP1, *A*_SS + BMI_/*A*_SS_ = 0.86 ± 0.05 in L2/3, 0.85 ± 0.06 in L4, and 0.81 ± 0.08 in L5; *n* = 4; *P* < 0.05). These results indicate that the initial amplitude reduction effect of SS on LFP was dominant over the additional effects of simultaneously administered SS and BMI (for SAMR1, *A*_SS_/*A*_ctl_ = 0.49 ± 0.15 in L2/3, 0.48 ± 0.09 in L4 and 0.52 ± 0.26 in L5; for SAMP1, *A*_SS_/*A*_ctl_ = 0.36 ± 0.12 in L2/3, 0.48 ± 0.17 in L4 and 0.46 ± 0.19 in L5; *P* < 0.05). Although the additional BMI application seemed to be less effective in SAMP1 compared with SAMR1, we found no significant differences between the SAM strains in terms of the simultaneous application of the two drugs. Additionally, in the SAMP1 mice, we observed a tendency for the additional BMI application to have a greater modulatory effect on the slower components of LFPs after the negative peaks in L2/3 and L5, compared with that seen in SAMR1 mice (Figures 11Bb–d). This suggests that the slow dynamics of evoked LFP responses returning to the baseline were affected by the disinhibition. In the washout condition, the waveforms of the LFP responses had not fully recovered to those in the pre-drug condition.

Next, in older SAM mice, we examined the disinhibitory effects of the GABA_B_ receptor antagonist CGP55845 (CGP) on local AC networks by recording stimulus-driven LFPs after application of CGP (Figure 10B). Although CGP administration slightly increased (compared with control) the average negative peak amplitudes (*A*_CGP_) of stimulus-driven LFPs in L2/3 and L4 for AC slices in SAMR1 (Figures 10Ba1–c1) and SAMP1 (Figures 10Ba2–c2) mice this difference was not significant (for SAMR1, *A*_CGP_/*A*_ctl_ = 1.23 ± 0.24 in L2/3, 1.22 ± 0.14 in L4, and 1.22 ± 0.13 in L5, *n* = 4, *P* > 0.10; for SAMP1 slices, *A*_CGP_/*A*_ctl_ = 1.12 ± 0.13 in L2/3, 1.11 ± 0.11 in L4, and 0.89 ± 0.18 in L5, *n* = 4, *P* > 0.10). We also examined stimulus-driven LFP responses when the GABA_B_ receptor antagonist was released along with SS. Following administration of SS alone, simultaneous application of the two drugs (SS and CGP) resulted in no significant changes in the negative peak LFP amplitudes (*A*_SS + CGP_) in both older SAMR1 and SAMP1 mice in any layers compared with amplitudes (*A*_SS_) in the SS alone condition (for SAMR1, *A*_SS + CGP_/*A*_SS_ = 1.08 ± 0.06 in L2/3, 0.99 ± 0.10 in L4, and 1.12 ± 0.08 in L5; *n* = 3; for SAMP1, *A*_SS + CGP_/*A*_SS_ = 1.21 ± 0.18 in L2/3, 1.01 ± 0.07 in L4, and 0.98 ± 0.09 in L5; *n* = 3; *P* > 0.10).

### Salicylate-Induced Effects on Current-Injected Firing in L4 Pyramidal Neurons

To examine SS-induced effects on individual L4 pyramidal neurons in young adult mice aged 6–11 weeks (in weeks, 7.5 ± 0.9 for SAMR1; 6.2 ± 0.2 for SAMP1; 10.6 ± 0.7 for C57BL/6J), we recorded firing responses to current injection using the whole-cell patch-clamp technique. A total of 20 pyramidal neurons (five neurons for SAMR1; four for SAMP1; 11 for C57BL/6J) in L4 were identified by their triangular somata and pia-oriented apical dendrites (Figures 12A,Ba; see “Materials and Methods” section). The cell distance from the pia matter ranged from 420 μm to 500 μm; in μm, 490 ± 50 for SAMR1, 468 ± 39 for SAMP1 and 508 ± 56 for C57BL/6J. For the individual L4 pyramidal neurons in the three strains, perfusion with salicylate at 1.4 mM significantly reduced neural firing induced by the 1-s step-current in all tested cells (Figures 12A,Bb). The extent of the reduction in SS-induced firing rate differed among individual cells, as summarized in Figure 12C. Moreover, in several cases, current-injected firing in pyramidal neurons was nearly extinguished after SS application (e.g., Figure 12Ab). Overall, SS superfusion decreased the firing rate and profoundly increased inter-spike intervals in the last several spikes during the current injection (Figures 12A,Bc). Thus, group data for all three strains indicate that the percentage of change in current-evoked firing rates in pyramidal neurons following SS application was statistically significant (38.6 ± 8.8%, *n* = 14, *P* < 0.01), and no strain differences were found (Figure 12). In the washout condition, firing rates were recovered to the level of the control (pre-SS) condition in all neurons, indicating that the SS-induced effects on firing rates were reversible. To characterize pyramidal neuron action potentials induced by the step-current injection in the three mouse strains, we focused on five parameters: RP, spike amplitude, after-hyperpolarizing potential, spike half width, and AR (Table 3). We found no significant changes among the five parameters, indicating that the major SS-induced effects changed the firing rates of individual cells only. Further, to compare membrane input resistance of L4 pyramidal neurons before (control), during (salicylate), and after (washout) salicylate superfusion, we carried out current-clamp recording in young C57BL/6J mice (*n* = 6 neurons in five mice). The result indicates that in five neurons out of six, the input resistance (*R*) was significantly reduced during the salicylate superfusion (*P* < 0.01 in five neurons; *P* > 0.10 in one neuron, neuron #1): in control, *R* = 473.3 ± 60.2 MΩ; during salicylate application, *R* = 355.5 ± 77.5 MΩ; in washout, *R* = 460.1 ± 63.4 MΩ (Figure 12D). In the five neurons with the decreased input resistance, the decrease was reversible following washout.

**Table 3:** Sodium salicylate (SS) effects on characteristic properties of membrane action potentials in L4 neurons for the three strains.

Strain	Condition	RP in mV	Amplitude in mV	AHP in mV	HW in ms	AR in %
SAMR1	Control	−69.1 ± 4.1	69.6 ± 4.6	9.7 ± 0.8	1.5 ± 0.1	35.6 ± 4.4
	SS	−68.9 ± 4.0	68.2 ± 5.5	10.7 ± 1.6	1.4 ± 0.1	34.1 ±4.8
	Washout	−71.5 ± 3.6	67.6 ± 5.5	10.9 ± 1.2	1.5 ± 0.1	34.4 ± 8.9
SAMP1	Control	−68.5 ± 2.5	65.8 ± 3.0	10.4 ± 0.8	2.1 ± 0.1	43.0 ± 8.0
	SS	−71.4 ± 1.9	61.9 ± 3.2	13.4 ± 0.9	1.8 ± 0.2	48.9 ± 3.2
	Washout	−70.1 ± 2.8	61.8 ± 3.8	11.4 ± 1.1	2.1 ± 0.2	51.6 ± 6.9
C57BL/6J	Control	−62.6 ± 2.9	67.2 ± 3.4	11.1 ± 0.9	2.6 ± 0.5	34.2 ± 7.7
	SS	−60.8 ± 3.7	59.7 ± 2.7	10.1 ± 0.8	2.7 ± 0.4	38.9 ± 11.8
	Washout	−63.3 ± 2.5	63.3 ± 2.1	10.3 ± 1.2	2.6 ± 0.4	37.2 ± 8.1

*Data are the mean ± SEM. Abbreviation: RP, resting potential; AHP, after-hyperpolarizing potential; HW, half width of a spike; AR, adaptation ratio (see “Materials and Methods” section)*.

In summary, these data indicate that 1.4 mM SS functionally and reversibly impaired firing activity only in L4 pyramidal AC neurons in three strains of young adult mice and reduced the membrane input resistance in adult C57BL/6J mice, while other basic firing properties remained unchanged.

## Discussion

The age-related properties of salicylate administration as a tinnitus animal model are still unclear. In this study, we conducted ABR recordings *in vivo* and extracellular MEA recordings of AC slices *in vitro* to examine cortical layer-dependent changes in stimulus-driven LFP responses following systemic salicylate administration in younger and older SAMP1 and SAMR1 mice. Overall, we found that characteristic salicylate-induced changes were associated with ABR thresholds and that the reduction in LFP responses in both SAM strains was cortical layer-dependent.

### ABR Properties in Younger and Older SAMP1 and SAMR1 Mice

SAMP1 mice exhibit a neurobiological phenotype of age-associated hearing impairment (Takeda et al., 1981; Saitoh et al., 1994; Hosokawa et al., 2013). Morphological studies have demonstrated an age-related decrease in both the size of spiral ganglion neurons and the cell density in SAMPl and SAMR1 mice (Saitoh et al., 1994). Additionally, changes of age-dependent loss in spiral ganglion neurons and hair cells appear earlier and progress more rapidly in SAMP1 vs. SAMR1 mice (Saitoh et al., 1994). Moreover, hearing impairment in SAMP1 is suggested to originate from a combination of sensory and strial (metabolic) presbycusis (Iwai et al., 2008), as well as neural presbycusis (Saitoh et al., 1994).

Generally, ABRs reflect the synchronous short-latency synaptic activity of successive nuclei along the peripheral afferent auditory neural pathway, including the cochlea and/or compound action potentials of auditory nerve cochlear nuclei, contralateral superior olivary complex, lateral lemniscus, and contralateral lateral IC (Henry, 1979; Parham et al., 2001; Galbraith et al., 2006). Saitoh et al. (1994) reported that for ABRs to tone stimuli with frequencies ranging from 2 kHz to 64 kHz, SAMP1 mice exhibited more rapid age-related hearing decline compared with SAMR1 mice over the age of 2 months. In contrast, we did not find significant differences of ABR threshold among the younger strains. Because we used click sound which included a wider range of frequencies in each stimulus, the type of sound stimulation could possibly cause the differences. In addition, their results show that ABR thresholds in 12-month-old SAMP1 mice are elevated to levels similar to those observed in 20-month-old SAMR1 mice, and that highly-sensitive frequencies (16 and 32 kHz) are no longer sensitive in 12-month-old SAMP1 and 20-month-old SAMR1 mice (Saitoh et al., 1994).

Here, the results we obtained are summarized as follows: (i) in both younger and older SAMR1 and SAMP1 mice, aging-related ABR properties among the SAM strains were not prominent at either age; (ii) among the SAM strains, amplitudes were significantly different in wave I in the younger group and wave III in the older group; and (iii) that younger vs. older mice in both SAM strains exhibited significantly different changes in wave II amplitude in response to pure tones with 65 dB SPL could reflect age-related differences in ABR threshold for small sounds. In the present study, we used a click sound stimulus with many frequency components to insure that there were no differences in ABR thresholds among the SAM strains. Overall, because more server hearing loss expected in SAMP1 than SAMR1 was not clearly observed, at least in our experimental setup using the click sound, the SAM strains were not necessarily suitable for a presbycusis animal model.

### SS-Induced Effects on LFP Responses Associated with ABR Threshold

Compared with the control condition, SS administration decreased stimulus-driven LFP responses in all layers, in all SAM AC slices (Figures 3, 4). Among the SAM strains, we found no significant differences in SS-induced LFP responses. However, in the older SAM mice, the amplitude ratios of LFP responses between the pre- and post-SS conditions were larger in L2/3 compared with those in L4 and L5 (Figure 4). Thus, the greater the peripheral auditory loss, the stronger the SS-induced reduction of LFP amplitude in the AC.

Previous studies have reported that SS has overall excitation effects in the dorsal cortex of the IC of adult rats (Patel and Zhang, 2014), the hippocampal CA1 of young rats (Gong et al., 2008), and the AC of young rats (Wang et al., 2006). In contrast, other studies have reported that reduction of neural excitability in fusiform cells of the dorsal cochlear nucleus (Wei et al., 2010) and decrease of excitatory synaptic transmission in the MGB of young rats (Su et al., 2009) and balanced firing rates of young rats (the arithmetic mean of changes in decreased and increased firing rates was nearly zero; Su et al., 2012). In particular, previous studies using electrophysiological recording in adult rats *in vivo* have found that local SS application in the AC generally reduces spontaneous activity yet enhances sound-driven activity (Lu et al., 2011).

The discrepancy between previous findings and those in the present study can be attributed to the use of a different experimental species (rat vs. mouse), recording technique (*in vivo* vs. *in vitro*) and animal ages (juvenile vs. adult). Particularly, most previous studies of *in vitro* brain slice preparations used very young animals (e.g., at ages of 13–21 postnatal days). Such results indicate that the effects of salicylate on neural activity are profoundly dependent on brain regions and the ages of examined animals. Also, populational activity of neurons in a local circuit is not necessarily determined by only results obtained at a single cell level. A combining method with different levels from single cells to local circuits should be needed to understand such widely varied salicylate effects on different auditory structures. In addition, we used slice preparations that were fully isolated from auditory nuclei in the auditory pathway and other brain regions that would be connected *in vivo*. However, local circuits and connections still remained to some extent in our AC slices. Our technique ensured that the SS-induced effects on neural activity were not modulated by other brain regions, but directly evoked by activity at the stimulation site.

To study the direct SS-induced effects on the local AC circuit, we associated peripheral properties observed in the ABR recordings with stimulus-driven LFP responses in the AC slice preparations. Particularly, to examine whether the SS-induced reduction of LFP peak amplitude was associated with the sensitivity of the auditory periphery in the tested SAM, we calculated correlation coefficients (*r*-values) between ABR thresholds and the ratio of peak amplitudes before and after SS superfusion (Figure 5). The results revealed a negative correlation between ABR thresholds and peak amplitude ratios in L2/3 and L4 for both strains and in L5 for SAMR1 only. Thus, in the present study, we found cortical layer-specific differences in SS-induced effects on stimulus-driven LFPs among the two SAM strains. Additionally, in the two SAM strains with a greater degree of hearing loss (ABR threshold ≥45 dB SPL), the SS-induced reduction of LFPs was significantly larger in L2/3 and L4 vs. L5 (Figures 5A,B). This is the first demonstration of AC-layer-specific SS-induced changes in aged mice. However, a previous study reported a stronger salicylate effect in L4 to L6 compared with L1 to L3 in the AC of younger mice (Basta et al., 2008).

### SS-Induced Effects on Current-Injected Firing in L4 Pyramidal Neurons

The evoked LFPs of the local network in the AC slices can be influenced by three possible factors: (i) intrinsic membrane properties of single neurons and (ii) inhibitory and/or (iii) excitatory synaptic transmission of activated responses in the local network.

A previous *in vitro* study of brain slice preparations reported that, for very young rats (12–19 postnatal days), perfusion with 1.4 mM SS had little effect on current-injected firing of L2/3 pyramidal neurons in AC slices (Su et al., 2009). They reported that during SS superfusion in a whole-cell *in vitro* patch-clamp recording preparation, the pyramidal neurons maintained a stable firing rate. The species (rat vs. mouse), cortical layer (L2/3 vs. L4) and age (juvenile vs. adult) we targeted were all different from those examined by Su et al. (2009); we examined SS-induced effects on L4 pyramidal neurons in the AC of young adult (6- to 11-week old) mice using whole-cell patch-clamp recording. However, we used the same SS concentration as Su et al. (2009), and found that, in the AC of young adult mice, SS functionally and reversibly impaired the firing rate and reduced membrane input resistance of L4 pyramidal neurons. The decreased membrane input resistance may induce the suppressive effect of SS on the synaptic responses of excitatory input and contribute to lower neuronal excitability as well as reducing the firing rate to the identical input in the presence of SS. Thus, excitatory L4 pyramidal neurons were less activated to the direct electric stimulation during SS administration.

Although the cellular mechanism underlying the SS-induced reduction of firing rate in L4 pyramidal neurons in the AC is still unclear, SS has been found to block voltage-gated sodium channels (Liu and Li, 2004; Liu et al., 2007) and L-type calcium channels (Liu et al., 2005) in the AC and IC. Further, at high doses, SS or aspirin can inhibit acid-sensing ion channels (Wang et al., 2012), which is likely to modulate the firing rate of neurons. Also, SS suppresses serotonin-induced enhancement of GABAergic spontaneous inhibitory postsynaptic currents in the rat IC *in vitro* (Wang et al., 2008) and modulates neural activity in the IC and AC *in vivo* (Liu et al., 2003). However, our present findings suggest that SS may directly modulate excitability of L4 pyramidal neurons.

In our recording experiments, we found it was very difficult to obtain stable recordings (over 40 min) of firing activity in response to current injection applied to single pyramidal neurons in older mice (over 12 weeks old). Thus, we were unable to explore the salicylate effects on firing properties among the individual older mouse strains, and have reported only the data obtained from young adult mice. The SS-induced effects on older SAM are still unknown.

### Effects of a GABAergic Agonist Administered Individually and with SS on LFP Responses

Previous studies have reported that electrophysiological changes in the AC during aging are associated with specific neurochemical changes related to γ-aminobutyric acid (GABA) neurotransmission (Mendelson and Ricketts, 2001), age-related loss of GABA_A_ receptors, and related auditory functional changes induced by GABA_A_-receptor dysfunction (Yu et al., 2006; Caspary et al., 2013; Brewton et al., 2016). The largest age-related changes (around 40% decrease relative to control) in glutamic acid decarboxylase messaging were found in the A1 L2 (Ling et al., 2005). During aging, modulation of GABAergic neurotransmission is expected to significantly alter spontaneous and stimulus-driven neural activity. Therefore, age-related decreases in GABAergic neurotransmission might influence activity in AC laminar profiles.

In the younger SAM strains, we found a positive correlation between ABR thresholds and the ratios of peak amplitudes before and after MSC superfusion (Figure 8). This differed from the corresponding results regarding SS-induced changes in the older SAM strains (Figure 5). The slopes of the correlations in the younger SAMR1 mice were steeper than those in the younger SAMP1 mice (Figure 8), indicating strain differences in the direct pharmacological effects of GABA_A_ receptors and implying that aging can decrease the correlation slopes. In contrast, we found no strain differences in older SAM mice, suggesting that the amplitude ratios between pre- and post-MSC were saturated.

In the present study, like SS, the GABA_A_ receptor agonist MSC significantly reduced stimulus-driven LFP responses in the SAM strains (Figure 6). Therefore, salicylate has a possibility to induce LFP reduction through effects on excitatory transmission and/or inhibitory transmission. In the SAM strains, to examine the amount of LFP peak amplitude decreased by MSC in the presence and absence of SS, we compared the two amplitude ratios A_MSC_/A_ctl_ (Figure 6) and A_SS + MSC_/A_SS_ (Figure 9). The result indicates that the differences of the ratios in all layers were not statistically significant in the two strains, suggesting that no interactions of SS and MSC appeared and that SS administration did not profoundly influence the GABAergic receptor-mediated changes in the evoked LFPs through MSC. This support that, with respect to the LFP reduction, the direct effects of salicylate on the excitatory transmission could be more dominant. Otherwise, this implies that the concentration of SS that we used did not maximally activate the GABA_A_ receptors.

However, previous studies reported that SS has been found to reduce the activity of fast-spiking interneurons (presumable inhibitory neurons releasing GABA) and suppress inhibitory postsynaptic currents in pyramidal neurons in L2/3 (neurons receiving GABAergic inputs) of very young (12−15 and 12−19 postnatal days) rats (Wang et al., 2006; Su et al., 2009). The reduction of inhibitory synaptic transmission can induce the increase of neural excitability of the local circuit, which contradicts our results obtained here. Further, in rat MGB neurons, salicylate simultaneously reduced the excitatory and inhibitory postsynaptic responses evoked by stimulating the brachium of the IC (Su et al., 2012). In the AC slice preparations examined here, we do not rule out the possibility that SS can modulate both excitatory and inhibitory synaptic transmissions so that they are likely to be balanced in the evoked LFP recording. The possibility will be tested in our future studies.

Further, the reduced later-phase CSD pattern that we observed in older SAMP1 mice (Figure 9Bb) might reflect the age-related disruption of GABA neurotransmission in L2/3 of the AC. Generally, CSD profiles reflect the activity components of synaptic inputs around the recorded area, rather than information output such as action potentials. Therefore, the observed suppression of the later-phase CSD pattern is likely associated with the mechanism by which AC neurons code sensory information, including the frequency and intensity of sound, in an age-dependent manner.

### Effects of a GABAergic Antagonist Administered with and without SS on LFP Responses

GABA_A_ antagonist (BMI) application has been found to elicit late-latency components in stimulus-driven LFPs in AC slices from young (three-week-old) rats (Shin et al., 2001) and young (6- to 10-week-old) mice (Alefeld et al., 1998). Further, some concentrations of BMI induced large LFP oscillations (Alefeld et al., 1998) as well as our obtained results. Similarly, we found that in AC slices from younger and older SAM mice, BMI elicited late-latency components with positive peaks in stimulus-driven LFPs.

Additionally, our results indicate that AC slices in younger mice were more sensitive to BMI than older mice, so that the large LFP oscillations in the slices from younger mice prevented us from the further recording during the BMI application over 40 min. Actually, in the LFPs of the younger mice, the late-latency component with a large positive peak 15 ms after from the stimulation onset gradually increased (see Figures 10Ab,c) and eventually surpassed the upper range of our voltage recording. Further, the more the time after BMI administration advanced, the smaller the amplitude of the fast negative-going peak became gradually. This result also shows that the BMI application contributed to reduce the negative-going peak and shifted the total LFP waveform upward (Figure 10A).

Generally, LFPs are an indicator of voltage changing (depolarizing or hyperpolarizing) events including active excitatory and inhibitory synaptic populations and axonal depolarization. Therefore, the late-latency components mainly reflected the voltage changing associated with the BMI-induced GABA_A_-related synaptic events. In contrast, the fast negative-going peaks were associated with the excitatory synaptic events as well as the GABA_A_-related synaptic events (Yamamura and Tateno, 2017). With regard to the older SAM strains, although the slices of SAMR1 were more likely to be sensitive to BMI than those of SAMP1, the differences of the negative-going peaks among the strains were not statistically significant (Figure 10A). However, the BMI sensitivity seemed to affect the amplitude of the slow positive peak of the late-latency component (Figures 10Ab,c).

To examine the disinhibitory effects of the GABA_A_ receptor antagonist on local AC networks, we recorded stimulus-driven LFPs when SS was released along with BMI. We found that simultaneous application of SS and BMI produced activity changes that suppressed large LFP oscillations, reflecting reduction of disinhibition by BMI. This may reflect that salicylate reduces inhibitory postsynaptic responses in the AC of the older mice, as previously reported in reduction of inhibitory postsynaptic responses of the rat MGB evoked by stimulating the brachium of the IC (Su et al., 2012) and suppression of inhibitory post synaptic currents in rat pyramidal neurons in L2/3, which receive GABAergic input from L4 (Wang et al., 2006). Also, in the current study, salicylate-induced effects on excitatory synaptic transmission were not directly examined at a cellular level. In the future, we plan to test the possibility of salicylate reduction of inhibitory postsynaptic responses as well as the excitatory synaptic transmission in the AC and will compare our results with those reported by the previous studies.

In addition, our results indicate that postsynaptic GABA_B_ receptors are unlikely to be major targets of SS. Activation of GABA_B_ receptors by a single brief synaptic input can result in inhibitory postsynaptic potentials from 50 ms to 800 ms after the onset of stimulation (Deisz et al., 1997). Application of CGP would be expected to result in disinhibition over a much longer time scale. However, we found no significant differences in LFP responses before and after CGP application. In addition, salicylate can possibly induce the presynaptic effects on evoked LFPs, and the possibility will be examined as one of our future challenges.

### Limitations and Implication of the Current Study

In the current study, only young adult mice (6–11 weeks old) were used for whole-cell patch-clamp recording, whereas extracellular MEA recordings of brain slice preparations were conducted in both younger and older mice. Because we obtained no stable patch-clamp recordings of older animals, we did not collect sufficient data for statistical analysis of data for this age group. Therefore, the selection of animal age fully depended on the recording technique, and was thus one of the limitations in the present study. Accordingly, the results obtained from young adult vs. older mice and those from whole-cell patch-clamp vs. extracellular MEA recordings should be interpreted with care.

The present study may throw light on the effect of aging on tinnitus in humans (Llano et al., 2012). Previous studies have reported that aging is related with decreases in cortical GABAergic transmission in animal models (Ling et al., 2005; Caspary et al., 2008; Burianova et al., 2009; Hughes et al., 2010). In addition, inhibition effect of salicylate on excitatory synaptic transmission may be associated with salicylate-induced spontaneous hypoactivity in AC *in vivo* (Yang et al., 2007). The salicylate-induced changes observed in our tinnitus model may involve interactions between reduced cortical excitatory and GABA_A_ergic transmission in mice with accelerated aging. Whether accelerated aging together with application of a tinnitus model have a greater combined effect on auditory cortical GABA levels is unknown. However, such a dual (two-factor) mechanism may help to explain the high percentages of tinnitus observed with aging (Møller, 2011). Further, if aging is accompanied by both differences in physiology and GABA sensitivity in the AC, potential therapeutic mechanisms for tinnitus may operate differently in young vs. aged subjects. Hence, scientists developing new therapeutic modalities for tinnitus treatment may benefit from using both aged and young animals in their translational research.

## Author Contributions

MN and AS: acquisition of data, analysis and interpretation of data, drafting and revising the article, final approval of the version to be published. TT: design of the experiments, analysis and interpretation of data, revising the article, final approval of the version to be published.

## Conflict of Interest Statement

The authors declare that the research was conducted in the absence of any commercial or financial relationships that could be construed as a potential conflict of interest. The reviewer S-JC and handling Editor declared their shared affiliation.

## Abbreviations

ABR: auditory brainstem response
AC: auditory cortex
ACSF: artificial cerebrospinal fluid
AHP: afterhyperpolarization
AR: adaptation ratio
A1: primary AC
BMI: bicuculline methiodide
CSD: current source density
GABA: γ-aminobutyric acid
IC: inferior colliculus
L: layer
LFP: local field potential
MEA: multielectrode array
MGB: medial geniculate body
MSC: muscimol
SAM: senescence-accelerated mice
SAMR1: senescence-resistant inbred strain
SAMP1: senescence prone inbred strains
SEM: standard error of the mean
SS: sodium salicylate
SPL: sound pressure level.
