# Supplementary material for: Salicylate-Induced Suppression of Electrically Driven Activity in Brain Slices from the Auditory Cortex of Aging Mice
Source: Front Aging Neurosci. 2017 Dec 12;9:395. doi: 10.3389/fnagi.2017.00395 (PMC5732918; doi:10.3389/fnagi.2017.00395)
Supplement: Supplementary file 1 [file Data_Sheet_1.pdf]

2-Dec-17

Salicylate-induced suppression of electrically driven activity of brain slice preparations in the auditory cortex of aging mice

Minoru NAMIKAWA, Ayaka SANO, and Takashi TATENO

Supplementary Material

Appendix

A.1. Current source density analysis

To detect characteristic spatiotemporal patterns in neural activity through laminarly organized networks in the AC, standard current source density (CSD) analysis was used for the easily identifiable evoked current sinks and sources (Haberly and Shepherd, 1973; Freeman and Nicholson, 1975; Mitzdorf and Singer, 1979). To calculate CSDs from LFP data recorded at multiple sites, we used the standard CSD method (Einevoll et al., 2013); i.e., the second spatial derivatives of LFPs were replaced with the corresponding spatial differences, and estimates of the CSDs were calculated (see below). Here, we assumed constant extracellular tissue conductivity and permittivity, as well as simultaneous nervous tissue activation along the measurement axis. Prior to CSD analysis, we also used a cubic spline data interpolation technique to estimate three additional points between the two original data points that were experimentally obtained. Additionally, in several of the electrodes in some experiments where the stimulation artifact was large, we estimated LFPs from those obtained from the two adjacent electrodes. However, this estimation result did not change the overall CSD pattern profiles. Thus, the spatial resolution at successive points became finer, and the interval between the points was 37.5 μm. Briefly, a simple one-dimensional CSD analysis was performed using LFP values at 29 points along the vertical axis, which was perpendicular to the cortical surface and layers of the AC. Along the vertical "on-line" stimulation sites, we applied the one-dimensional CSD analysis to the estimated LFPs at each point and obtained the CSD using the following formula:

$$\mathrm{CSD}(z,t) \approx -\sigma\left[\varphi(z-\Delta z,t) - 2\varphi(z,t) + \varphi(z+\Delta z,t)\right] / (\Delta z)^2 , \quad (1)$$

where z is the spatial coordinate in the laminar direction, t is time, σ is conductivity of the extracellular space, φ is the LFP at z, and Δz is the inter-point interval (i.e., $\Delta z = 37.5$ μm). Note that two-dimensional CSD analysis and more advanced CSD analysis (Pettersen et al., 2006) provide similar CSD values at corresponding points as the one-dimensional analysis if the LFP changes horizontally adjacent to the target point at z (i.e., first derivatives in the horizontal axis) are similar to the target point. This was generally the case for the current data. Therefore, we used a one-dimensional CSD analysis to reduce the calculation time and cost. Furthermore, for simplicity, we assumed that σ was uniform and had a constant value ($\sigma = 0.30$ mS/mm) over space for all recording sites (Bedard and Destexhe, 2011). We repeated the same trials 10 times under identical conditions for each experiment, so that the CSD values represented the average over the trials. With respect to neural activity patterns, in general, current sinks

are interpreted to indicate depolarizing events such as active excitatory synaptic populations and axonal depolarization, i.e., indicating inward, usually excitatory, synaptic current flow (Mitzdorf, 1985; Telfeian and Connors, 1999). Current sources, in contrast, reflect passive return currents most of the time, indicating indicates outward, usually passive, current flow.

Figure legends

Supplementary Figure S1. Among the four mouse groups (younger and older SAM-R1 and -P1), two characteristic properties (latency and half width) of stimulus-driven LFPs in the three layers (L2/3, L4, and L5) before and after the SS superfusion are shown. A, Latencies (L_{ctl} and L_{SS}) in L2/3 (black), L4 (white), and L5 (gray) among the mouse groups were plotted under the control condition in (a) and the 1.4 mM SS superfusion condition in (b), respectively. The ratios of the latencies (L_{SS} /L_{ctl}) among the four mouse groups were also illustrated in (c). B, Similarly, half widths (HW_{ctl} and HW_{SS}) in L2/3 (black), L4 (white), and L5 (gray) among the mouse groups were plotted under the control condition in (a) and the 1.4 mM SS superfusion condition in (b), respectively. The ratios of the half widths (HW_{SS} / HW_{ctl}) among the four mouse groups were also illustrated in (c). Error bars represent standard error of the mean (SEM). Data are shown in mean ± SEM.

Supplementary Figure S2. Among the four mouse groups (younger and older SAM-R1 and -P1), two characteristic properties (latency and half width) of stimulus-driven LFP responses before and after the MSC superfusion are shown. A, Latencies (L_{ctl} and L_{MSC}) in L2/3 (black), L4 (white), and L5 (gray) among the mouse groups were plotted under the control condition in (a) and the 5-µM MSC superfusion condition in (b), respectively. The ratios of the latencies (L_{MSC} / L_{ctl}) among the four mouse groups were also illustrated in (c). B, Similarly, half widths (HW_{ctl} and HW_{MSC}) in L2/3 (black), L4 (white), and L5 (gray) among the mouse groups were plotted under the control condition in (a) and the 5-µM MSC superfusion condition in (b), respectively. The ratios of the half widths (HW_{MSC} /HW_{ctl}) among the four mouse groups were also illustrated in (c). Error bars represent standard error of the mean (SEM).

Supplementary Figure S3. Morphology and characteristic firing of L4 pyramidal neurons and fast-spiking interneurons in the AC of adult C57BK/6J mice. A, L4 pyramidal neurons. In (a), a morphological trace of a typical pyramidal neuron was shown (see Methods). In (b), action potential trains evoked by a 1-s depolarizing current (150 pA) in a pyramidal neuron. In (c), the waveform of the third action potential of the same neuron in (b). In (d), relationships of firing rates vs. injected currents in two pyramidal neurons (PN1 and PN2). B, L4 fast-spiking interneurons. Similarly, in (a), a morphological trace of a typical interneuron was shown. In (b), action potential trains evoked by a 1-s depolarizing current (150 pA) in an interneuron. In (c), the waveform of the third action potential of the same neuron in (b). In (d), relationships of firing rates vs. injected currents in two fast-spiking interneurons (FS1 and FS2).

References

Bedard C, Destexhe A (2011) Generalized theory for current-source-density analysis in brain tissue. Phys Rev E Stat Nonlin Soft Matter Phys 84:041909.

Einevoll GT, Kayser C, Logothetis NK, Panzeri S (2013) Modelling and analysis of local field potentials for studying the function of cortical circuits. Nat Rev Neurosci 14:770-785.

Freeman JA, Nicholson C (1975) Experimental optimization of current source-density technique for anuran cerebellum. J Neurophysiol 38:369-382.

Haberly LB, Shepherd GM (1973) Current-density analysis of summed evoked potentials in opossum prepyriform cortex. J Neurophysiol 36:789-802.

Mitzdorf U (1985) Current source-density method and application in cat cerebral cortex: investigation of evoked potentials and EEG phenomena. Physiol Rev 65:37-100.

Mitzdorf U, Singer W (1979) Excitatory synaptic ensemble properties in the visual cortex of the macaque monkey: a current source density analysis of electrically evoked potentials. J Comp Neurol 187:71-83.

Pettersen KH, Devor A, Ulbert I, Dale AM, Einevoll GT (2006) Current-source density estimation based on inversion of electrostatic forward solution: effects of finite extent of neuronal activity and conductivity discontinuities. J Neurosci Methods 154:116-133.

Telfeian AE, Connors BW (1999) Epileptiform propagation patterns mediated by NMDA and non-NMDA receptors in rat neocortex. Epilepsia 40:1499-1506.

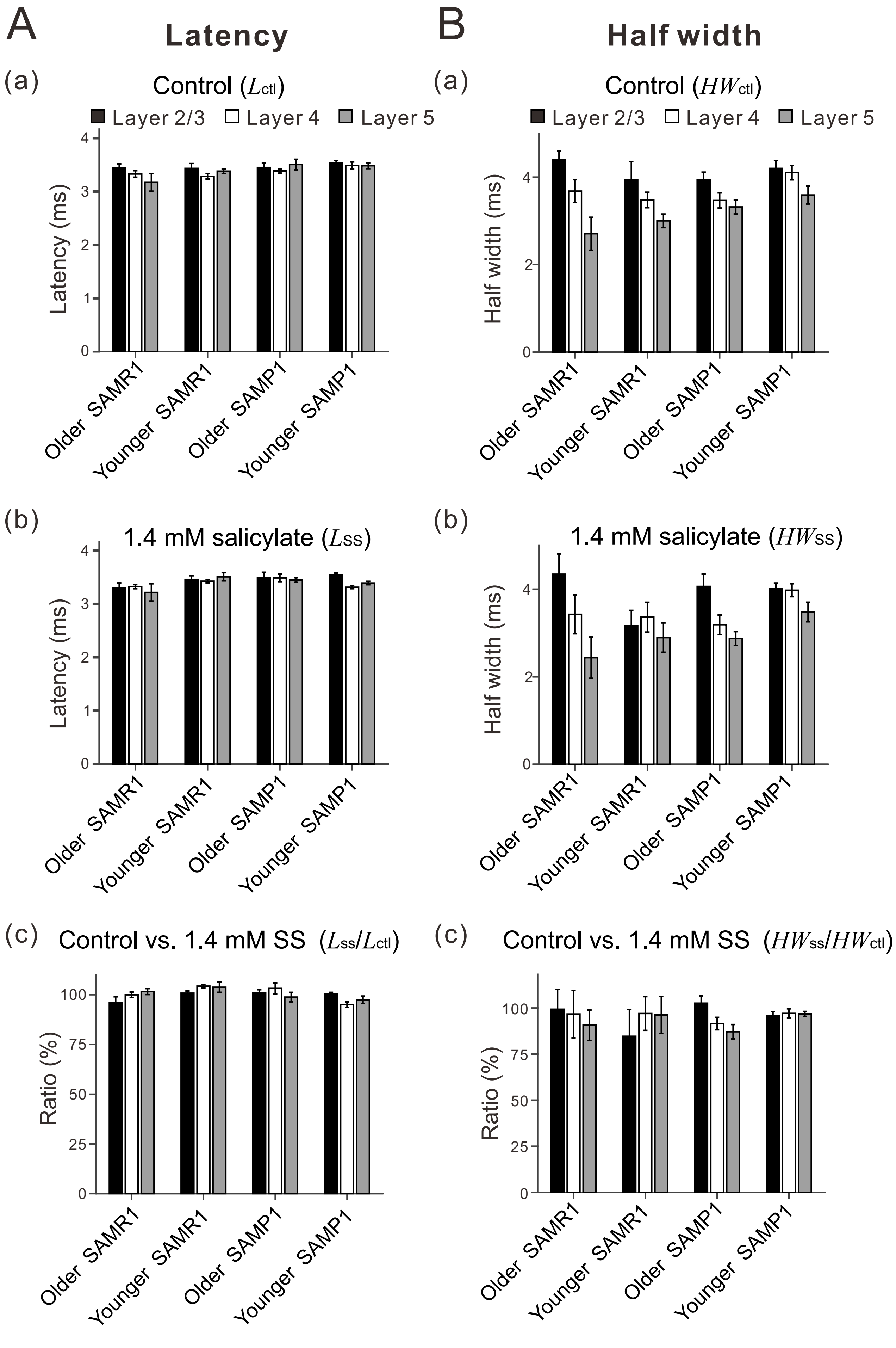

Supplementary Figure S1
Namikawa et al., 2017.12.02

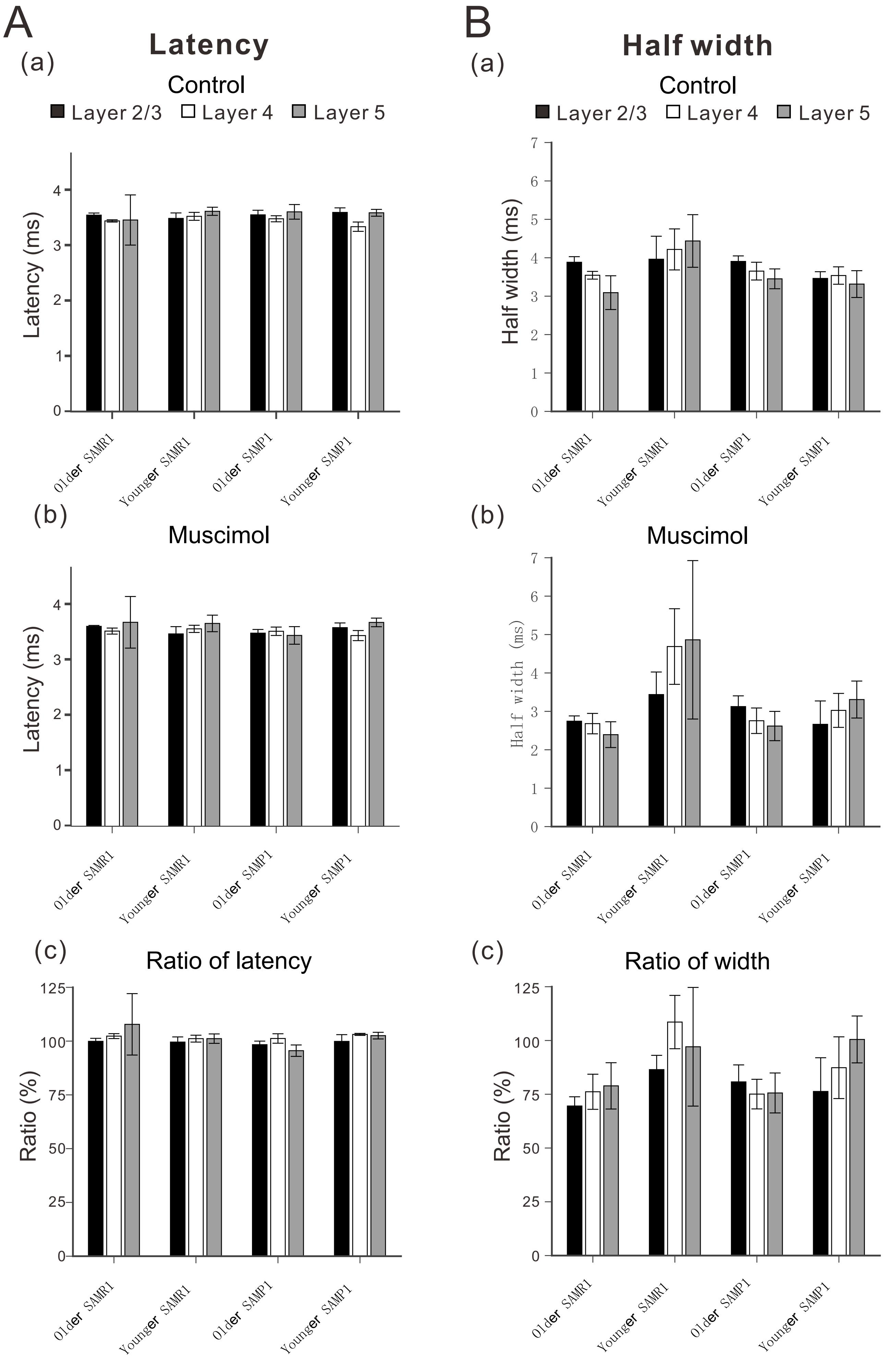

Supplementary Figure S2
Namikawa et al., 2017.12.02

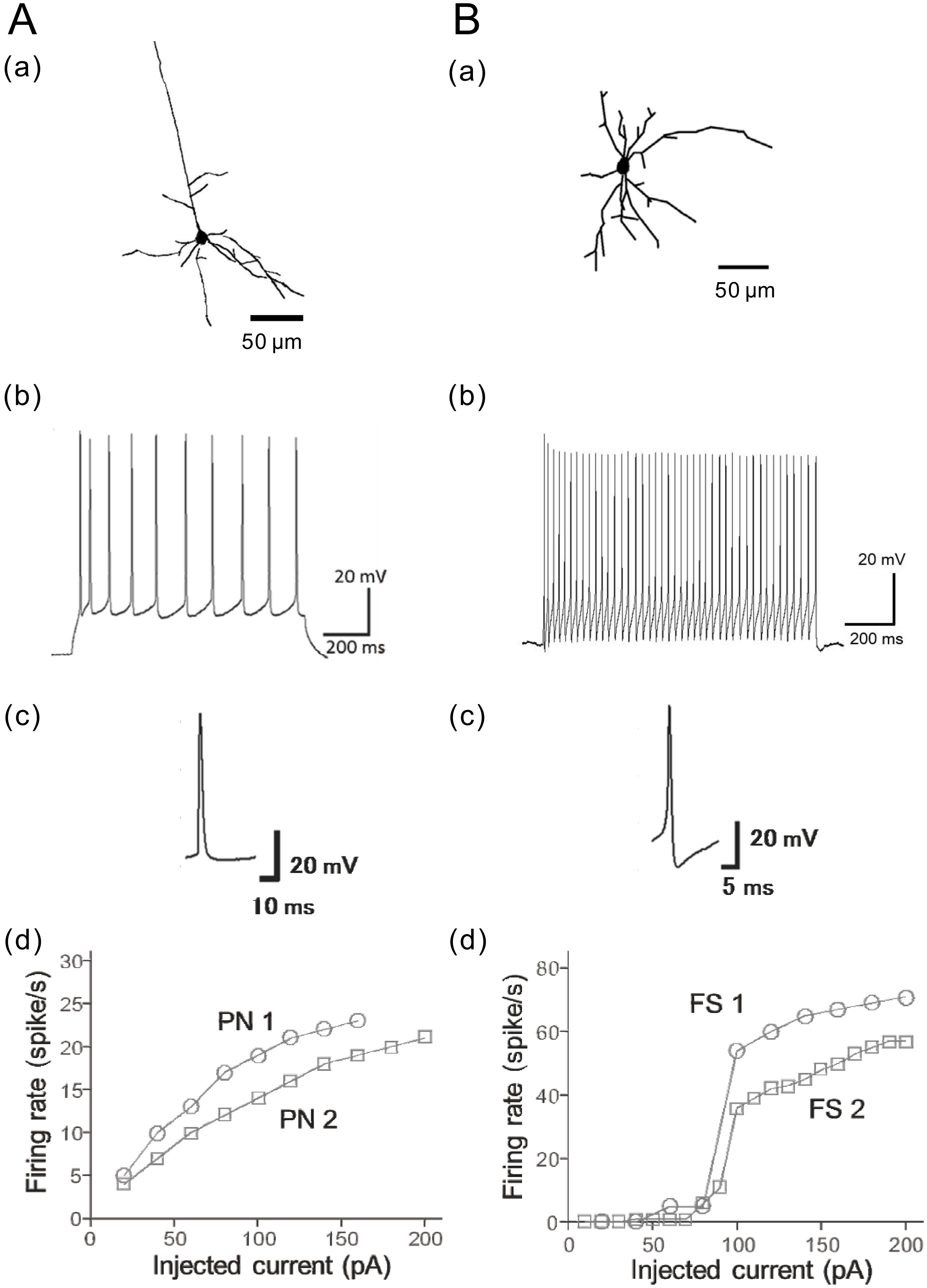

Supplementary Figure S3,
Namikawa et al., 2017.12.02
